# Effects of intake of four types of snack with different timings on postprandial glucose levels after dinner

**DOI:** 10.1007/s00394-023-03138-4

**Published:** 2023-04-15

**Authors:** Hirofumi Masutomi, Yui Mineshita, Katsuyuki Ishihara, Kazuko Hirao, Shigenobu Shibata, Akiko Furutani

**Affiliations:** 1 Calbee, Inc. Research and Development Division, Utsunomiya, Tochigi 321-3231 Japan; 2 Laboratory of Physiology and Pharmacology, School of Advanced Science and Engineering, Waseda University, Shinjuku, Tokyo 162-0056 Japan; 3 Faculty of Home Economics, Aikoku Gakuen Junior College, Edogawa-ku, Tokyo 133-8585 Japan; 4 Graduate School of Advanced Science and Engineering, Waseda University, Shinjuku-ku, Tokyo 162-0056 Japan

**Keywords:** Glucose, Snack, Second-meal effect, Potato chips, Granola, Black bean, Sweet potato

## Abstract

**Purpose:**

It has been reported that the consumption of fruit granola (FG), mulberry leaves, and barley cookies as an afternoon snack suppresses the postprandial increase in glucose levels at dinner. However, there have been no reports on the second-meal effect of snacking on popular snacks, such as potato chips (PC), roasted sweet potato (SP), and black beans (BB), or on the interval between snacking and dinner.

**Method:**

The present study was an open-label randomized crossover trial of five study groups (PC, SP, BB, FG, and no snack) regarding the second-meal effects with different intervals between snacks and dinner. The subjects consumed prescribed meals for lunch and dinner at 12:00 and 19:00, and a snack fixed at 838 kJ (= 200 kcal) at 15:00 or 17:00.

**Results:**

When the participants snacked at 15:00, the postprandial glucose elevation at dinner was suppressed in the FG and SP groups, and the area under the curve (AUC) was also low. When they snacked at 17:00, the postprandial glucose elevation was suppressed in all the groups. The AUCs for PC, FG, and SP were lower than those for no snacking. On the other hand, carbohydrate intake increased with snacking, but the total AUC of snacks and dinner did not differ in any of the groups. The duration of hyperglycemia decreased with snack intake, as did the glucose amplitude.

**Conclusion:**

We believe that the intake of carbohydrates and soluble fiber in snacks is an important factor in the second-meal effect at dinner. These results will contribute to the development of snacking and research into the second-meal effect.

**Supplementary Information:**

The online version contains supplementary material available at 10.1007/s00394-023-03138-4.

## Introduction

When blood glucose spikes occur, insulin is secreted and the conversion of carbohydrates into fat is excessive, leading to obesity [1]. According to the International Diabetes Federation guidelines for postprandial glycemic control, postprandial and postload hyperglycemia is a risk factor for vascular disease [2]. In addition, the suppression of blood glucose spikes has been implicated as an important factor in vascular disease, as it affects prognosis [3]. In addition, prolonged fasting is known to be a factor in blood glucose spikes [4]. Previous studies have examined whether the postprandial blood glucose levels after dinner are modified by snacking, and have shown that eating snacks between lunch and dinner results in a slower increase in blood glucose levels after dinner than no snacking, as a second-meal effect [5].

The second-meal effect is a concept proposed by Jenkins et al. in 1982 [6]. It is the idea that the first meal also affects the postprandial blood glucose level after the second meal [7]. In recent years, some studies have reported the application of this second-meal effect to snacking. In 2018, it was reported that snacking on cookies at 15:30 and consuming dinner at 19:30 in type 2 diabetes (T2D) subjects suppressed the post-dinner blood glucose elevation [5]. Similarly, in 2019, it was reported that snacking on cookies at 15:30 and consuming dinner at 19:30 in healthy women suppressed the post-dinner blood glucose elevation [8]. The authors reported that eating mulberry leaves or barley grass powder with 156.6 kcal cookies at 17:00 in healthy subjects reduced the post-dinner blood glucose level at 21:00 [9]. In addition, a study in elderly people reported that snacking on a 154 kcal cookie containing 9.2 g of dietary fiber at 15:30 suppressed the post-dinner increase in blood glucose at 18:30 compared to a 154 kcal control cookie [10]. Our study using fruit granola, a type of cereal, as the test food showed that snacking on 114 kcal of fruit granola at 17:00 suppressed the postprandial increase in blood glucose levels after dinner at 20:00 and reduced the number of awakenings during sleep in healthy women [11].

One of the most popular snack foods is potato chips, which are produced by deep-frying thin slices of fresh potato [12]. The glycemic index (GI) of potato chips has been reported to be about 60 [13]. Roasted sweet potatoes are one of the foods unique to East Asia [14, 15]. The raw material, sweet potato, is considered a low-GI food, with a GI of 55 [16]. However, the GI of sweet potatoes varies depending on how they are cooked, and the GI of roasted sweet potatoes is estimated to be around 80 [17]. Studies in animal and human models suggest that sweet potatoes may play a role in blood sugar control [18]. Beans are a low-GI food and rich in vegetable protein [19]. In recent years, snacks made from beans have been developed as alternative snacks [20]. There are reports that black bean consumption improves insulin resistance by modifying the intestinal microflora, in addition to suppressing regular blood glucose levels [21]. Granola is a general term for several types of cereals, including oats mixed with syrup and baked, and is eaten in Europe, the USA, and Japan. According to a systematic review on oat cereals, the mean GI for granola and muesli across 27 reports was 56 (range 39–70) [22]. When granola was served as breakfast, postprandial blood glucose and insulin levels were suppressed [23].

There have been few studies on snacking foods that are widely distributed and consumed by the general public. In addition, the energy intake from snacks has not been standardized. In the present study, we examined the effects of snacking on potato chips, black beans, roasted sweet potatoes, and fruit granola, which are commonly consumed by Japanese people, on the second-meal effect at dinner. In many studies, only one point of time is set for snack intake and then determining the second-meal effect. However, people may eat snacks at earlier times before dinner or near to dinner. Therefore, in the current experiments, the snacks were consumed at 15:00 or 17:00.

## Materials and methods

### Study method

In this study, we defined “snacks” as the food eaten at 15:00 or 17:00 (Table 1). We prepared four snacks of fried black beans, potato chips, fruit granola (Calbee Inc., Tokyo, Japan), and roasted sweet potato (Potato Kaitsuka Ltd., Ibaraki, Japan) (Supplemental Figure S1). According to the Ministry of Health, Labor and Welfare, snacking is generally recommended to be under 200 kcal [24]. All four snacks had consistent energy counts of 838 kJ (= 200 kcal) (Table 2). We targeted for recruitment in this experiment a working, healthy generation who were more likely to snack between lunch and dinner.

**Table 1:** Test schedule

Time of meal	15:00/17:00	19:00
NO	–	Dinner
BB	Fried black beans	Dinner
PC	Potato chips	Dinner
FG	Fruit granola	Dinner
SP	Roasted sweet potato	Dinner

Study of 15:00 snack (N = 17)

Study of 17:00 snack (N = 16)

*NO* no-snack, *BB* fried black beans snack, *PC* potato chips snack, *FG* fruit granola snack, *SP* roasted sweet potato snack

**Table 2:** Nutrition information for snack

Group	BB	PC	FG	SP
Snack	Fried black beans	Potato chips	Fruit granola	Roasted sweet potato
Weight (g)	38.5	35.7	45.2	122.7
Energy (kJ)	838	838	838	838
Protein (g)	12.3	1.8	3.7	1.7
Fat (g)	13.3	12.9	7.1	0.2
Total carbohydrates (g)	10.8	19.4	32.7	47.9
Sugars (g)	4.5	17.9	28.3	43.6
Dietary fiber (g)	6.3	1.5	4.4	4.3
Soluble dietary fiber (g)	0.5	0.4	2.8	1.8
PFC balance				
Protein (% energy)	25.0	3.7	7.1	3.4
Fat (% energy)	58.7	57.0	31.4	0.9
Carbohydrates (% energy)	15.1	37.8	61.6	92.6

All four snacks had consistent energy counts of 838 kJ (= 200 kcal)

The PFC balance was calculated as follows: protein, calculated as the sum of amino acid residues = 17 kJ, protein (% energy) = ([g] * [17 kJ/g])/total energy; fatty acids, expressed in triacylglycerol equivalents = 38 kJ, fat (% energy) = ([g] * [38 kJ/g])/total energy; carbohydrate, available, calculated by difference = 17 kJ, dietary fiber, total = 8 kJ, carbohydrates (% energy) = ([sugars, g] * [17 kJ/g] + [dietary fiber, g] * [8 kJ/g])/total energy

STANDARD TABLES OF FOOD COMPOSITION IN JAPAN - 2020 - (Eighth Revised Edition) was referenced

*BB* fried black beans snack, *PC* potato chips snack, *FG* fruit granola snack, *SP* roasted sweet potato snack

The subjects were recruited at Aikoku Gakuen Junior College and selected as follows: (1) healthy persons aged 18 years and older; (2) persons who provided written consent for participation. Excluded from the study were (1) individuals who were allergic to the food used in the study; (2) individuals with glucose intolerance (abnormal at medical examination for the past 2 years); (3) individuals with fasting blood glucose > 110 mg/dL; (4) individuals with body mass indices (BMIs) > 30; (5) patients with severe liver disease, kidney disease, heart disease, lung disease, psychiatric disease, blood disease, etc.; (6) individuals who took drugs such as antihypertensive drugs; (7) those who wished to become pregnant or were pregnant/breastfeeding during the study; and (8) those who were judged by the study supervisor to be inappropriate for participation.

**Fig. 1:**
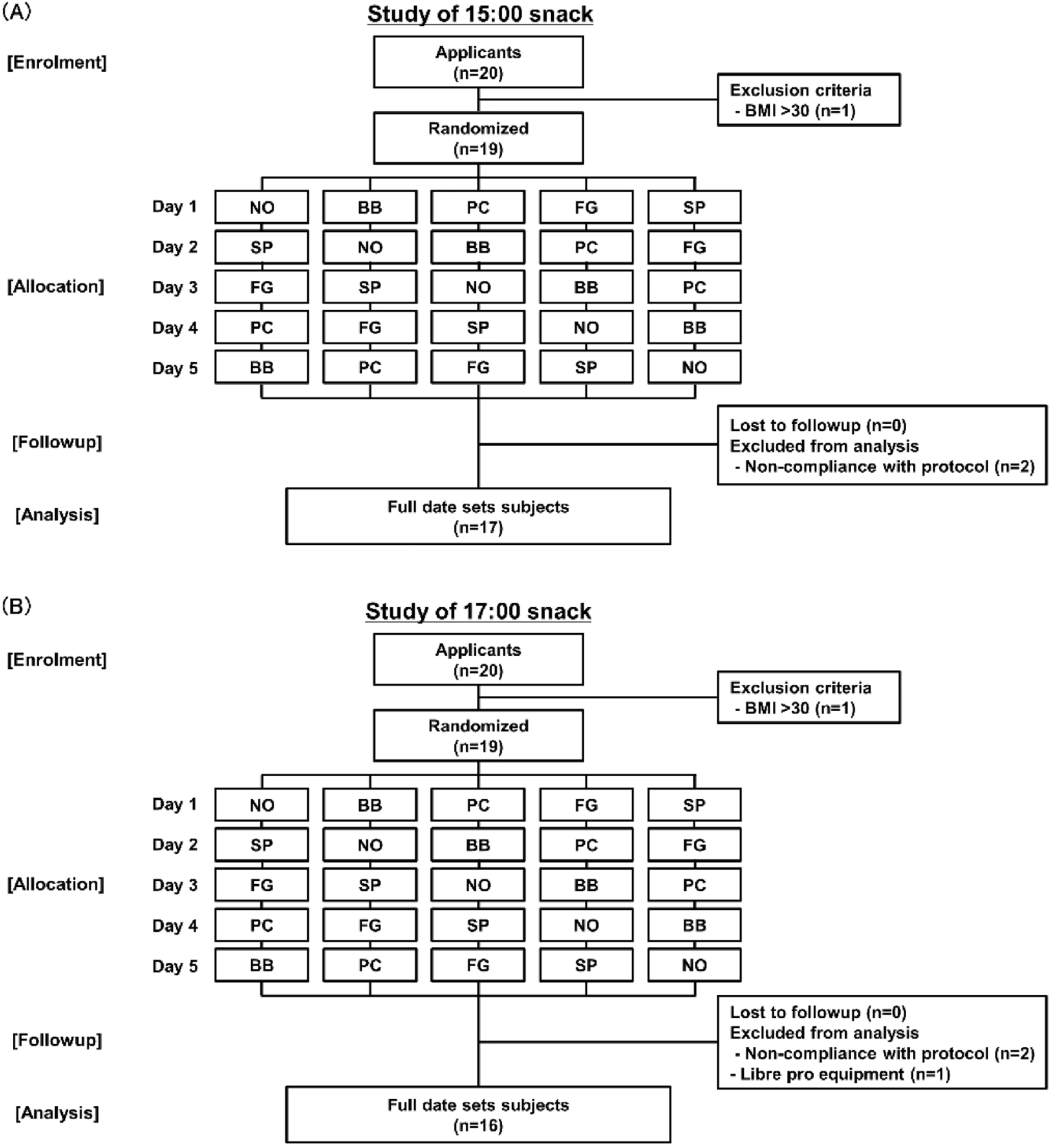
Study design. Randomization and screening of subjects for 15:00 snack (**A**). Randomization and screening of subjects for 17:00 snack (**B**). *NO* no-snack, *BB* fried black beans snack, *PC* potato chips snack, *FG* fruit granola snack, *SP* roasted sweet potato snack, *BMI* body mass index

Publicly recruited subjects were randomly assigned for the study methods. We set five randomized crossover trials (no-snack (NO), fried black bean snack (BB), potato chip snack (PC), fruit granola snack (FG), and roasted sweet potato snack (SP)) (Fig. 1A, B, Supplemental Table S1). The present study was an open-label randomized crossover trial of five study groups (PC, SP, BB, FG, and no snack) regarding the second-meal effects with different intervals between snacks and dinner. The subjects were randomized by M.K., who was not involved in this study. Glucose (Nippon Garlic Co, Gunma, Japan) was prepared as a snack for additional testing. The glucose was weighed, and 59.1 g was dissolved in 300 mL of water (Suntory, Tokyo, Japan) to be provided to subjects. The subjects ate lunch at 12:00, a snack at 15:00 or at 17:00, and dinner at 19:00 (Table 1). Lunch and dinner were prepared as prescribed meals. For lunch, white bread (Yamazaki Baking co., ltd, Tokyo, Japan), four kinds of main dishes (hashed beef, butter chicken curry, beef curry, and cream stew) (Nichirei foods Inc., Tokyo, Japan), and two kinds of soup (minestrone and corn soup) (Nichirei) were prepared as prescribed lunch (Supplemental Table S2). For dinner, rice (Sato foods Co., Ltd., Niigata, Japan), three kinds of main dishes (hamburger steak set, cheese in hamburger steak set, and fried chicken set) (Nichirei), and two kinds of soup (miso soup and corn cream soup) (Marukome, Tokyo, Japan; Cunol, Tokyo, Japan) were prepared as prescribed dinner (Supplemental Table S3). Supervision and investigation confirmed that the subject completed the lunch, snack, and dinner. The subjects wore glucose meters (Freestyle Libre Pro; Abbott Japan LLC, Tokyo, Japan) on the first day of the study. An over-1-day observation period was set for glucose monitoring stabilization [25, 26]. The foods permitted were lunch, snacks, and dinner, and the subjects were fasting from other meals from lunch until the next morning. The only drink allowed was water; other food and beverages were banned from lunch to the next morning.

The study protocol was registered with the University Hospital Health Information Network Clinical Trials Registry System (UMIN-CRT, registration number: UMIN000045006). This study was conducted with respect for the human rights of the participants, based on the Declaration of Helsinki, and with approval from the Ethics Review Committee of Aikoku Gakuen Junior College (approval number: 2019-R04). The subjects were verbally informed of the outline, purpose, investigation content, protection of personal information, etc., for this study and gave written consent. In doing so, they were also told that all the data obtained were coded and individuals could not be identified, that the data were analyzed in a population, that they would not be used for purposes other than research, that the subjects would not be disadvantaged, and that they would be free to participate in the research or not.

### Glucose levels

The glucose levels of the subjects were monitored using FreeStyle Libre Pro, an instrument capable of measuring interstitial glucose levels every 15 min for 14 consecutive days. The blood glucose levels from fingertips and interstitial blood glucose levels were reported to be correlated [27]. Another report also reported that Freestyle Libre Pro was highly correlated with capillary blood glucose levels, with a correlation coefficient of 0.95 (range 23–498 mg/dL) [28]. The FreeStyle Libre Pro device was placed on the upper arm of the subject by a physician. After the completion of the study, the devices were retrieved and data were read by a dedicated device. Three-item analyses were conducted: (1) the glucose level every 15 min after snack and dinner consumption; (2) the maximal glucose level; and (3) the area under the curve (AUC). The glucose levels were monitored and analyzed every 15 min from 12:00 to 24:00. The maximum glucose level for the snack was analyzed after 2 h. The AUC of the snacks was accumulated for 2 h from 15:00 to 17:00 or 17:00 to 19:00. The maximum glucose level for dinner was analyzed from 19:00 to 24:00. The AUC for dinner was accumulated for 5 h from 19:00 to 24:00. The total AUC was defined as the sum of the AUC after the snack and the AUC after dinner. The AUC was calculated using the trapezoidal method [11].

To observe the range of glucose variability, the amplitude range of glucose was defined as the difference between the maximum and minimum glucose during glucose monitoring. The glucose levels were categorized into three groups— < 70, 70–140, and > 140 mg/dL or higher—and a figure was created during glucose monitoring. Less than 70 mg/dL causes hypoglycemic symptoms [29]. A glucose spike indicates a blood sugar level of 140 mg/dL or higher [30, 31]. In this study, less than 70 mg/dL was considered hypoglycemic and more than 140 mg/dL was considered hyperglycemic.

### Visual analog scale (VAS) of hunger

Hunger until dinner was assessed using the visual analog scale (VAS) method with a scale of 100 mm [32]. The subjects were asked to fill in the form every hour from 13:00 to 19:00, and the distance from the edge (mm) was measured.

### Statistical analysis

A two-way repeated ANOVA was used to analyze the hourly glucose data and VAS, and Dunnett's test was used for multiple comparisons. A one-way repeated ANOVA was used for the analysis of the maximal glucose, AUC, and total AUC, and Dunnett’s test was used for multiple comparisons. The correlation used Spearman’s rank correlation coefficient. The amplitude range of glucose and spent time for glucose were determined by Friedman ranking, and the Dunn method was used for multiple comparisons. We used G*Power to determine the sample size. GraphPad Prism 8.4.3 was used as the statistical software for analysis, and the level of statistical significance was set at *p* < 0.05.

## Results

### Screening

In the study of the 15:00 snack, among the participants who volunteered to participate, 20 agreed. The internal exclusion criterion, a BMI > 30, excluded one individual (Fig. 1A). Two subjects were excluded from the analysis because they could not follow the protocol. The full data set consisted of 17 subjects, and the analysis was conducted. There were eight men and nine women, with a mean age of 37.9 ± 12.9 years (range 22–64 years) and BMIs of 21.4 ± 2.7 (range 17.0–25.9). There were no adverse events in these studies.

In the study of the 17:00 snack, among the participants who volunteered to participate, 20 agreed. The internal exclusion criterion, a BMI > 30, excluded one individual (Fig. 1B). Two subjects were excluded from the analysis because they could not follow the protocol, and one subject was missing data from Libre Pro. The full data set consisted of 16 subjects, and the analysis was conducted. There were nine men and seven women, with a mean age of 37.1 ± 12.3 years (range 23–64 years) and BMIs of 22.2 ± 2.7 (range 17.7–26.6). There were no adverse events in these studies either.

### Glucose levels at 15:00 snack

**Fig. 2:**
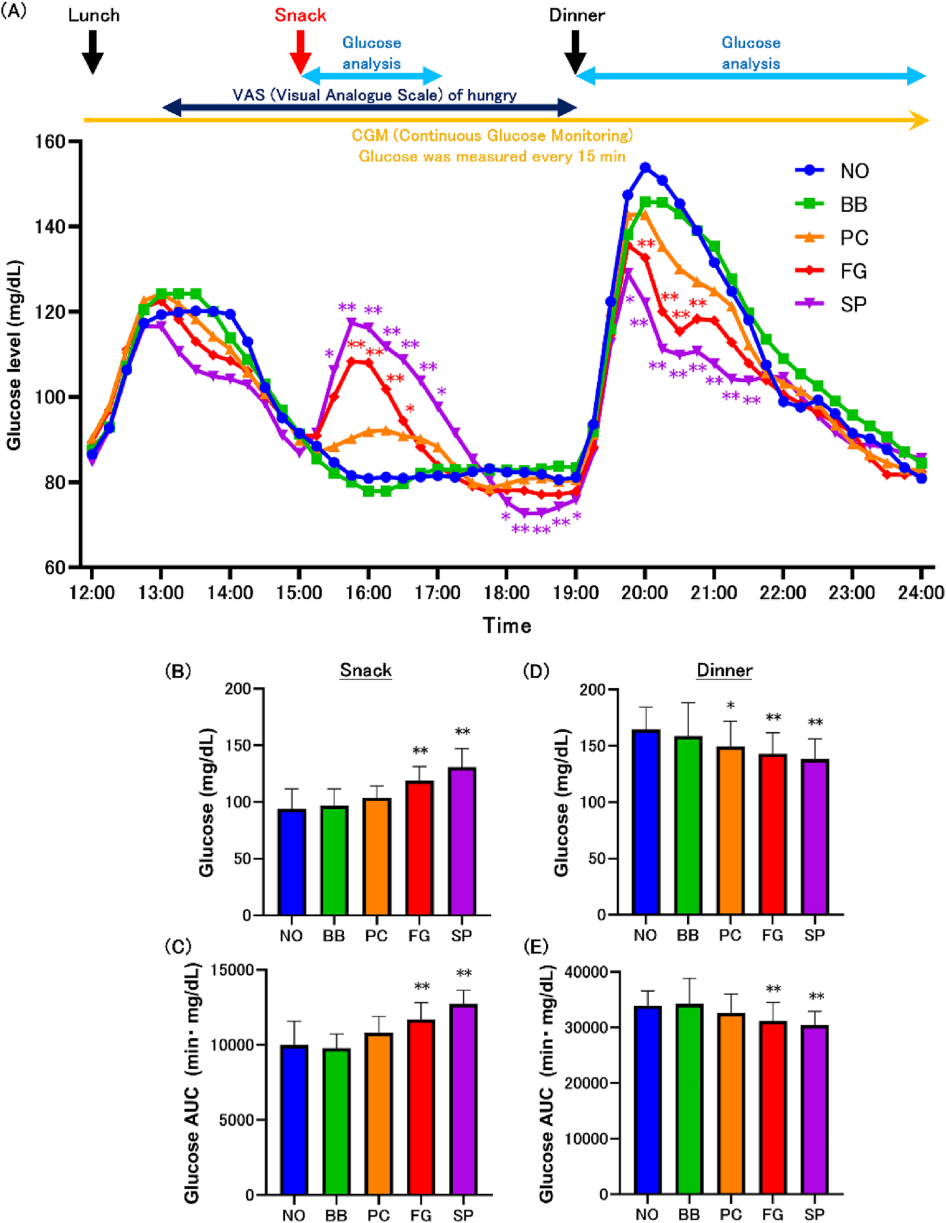
Response of glucose levels at 15:00 snack. Monitoring of glucose levels at 15:00 snack (**A**). Maximum glucose after snack intake (**B**). Area under the curve (AUC) of glucose levels after snack intake (**C**). Maximum glucose after dinner intake (**D**). AUC of glucose levels after dinner intake (**E**). Values are means ± standard deviations. Statistics vs. NO group, *p < 0.05, **p < 0.01. *NO* no-snack, *BB* fried black beans snack, *PC* potato chips snack, *FG* fruit granola snack, *SP* roasted sweet potato snack

When we examined the changes in glucose levels during the snack at 15:00, the postprandial blood glucose levels in the FG and SP groups were elevated (Fig. 2A, Supplemental Table S4). Compared with the change in maximal glucose level during the snack at 15:00, FG and SP groups showed increases in maximal glucose levels compared with NO group (Fig. 2B, Supplemental Table S5). The AUC similarly increased in the FG and SP groups (Fig. 2C, Supplemental Table S5).

When analyzing the glucose level variability at dinner, the pre-dinner glucose level in SP group was lower than that in NO group at 19:00 (Fig. 2A, Supplemental Table S4). The postprandial glucose level in the FG group was lower than that in the NO group at 20:00, 20:15, 20:30, and 20:45. The postprandial glucose level in the SP group was lower than that in the NO group at 19:45, 20:00, 20:15, 20:30, 20:45, 21:00, 21:15, and 21:30. Compared with the change in maximal glucose level during the dinner, PC, FG, and SP groups showed suppressed increases in maximal glucose levels compared with NO group (Fig. 2D, Supplemental Table S5). The AUC after dinner similarly showed a suppressed increase in the FG and SP groups (Fig. 2E, Supplemental Table S5). In the optional study, the postprandial glucose level at dinner in the glucose snack group was higher than that in the NO group (Supplemental Figure S2A).

### Glucose levels at 17:00 snack

**Fig. 3:**
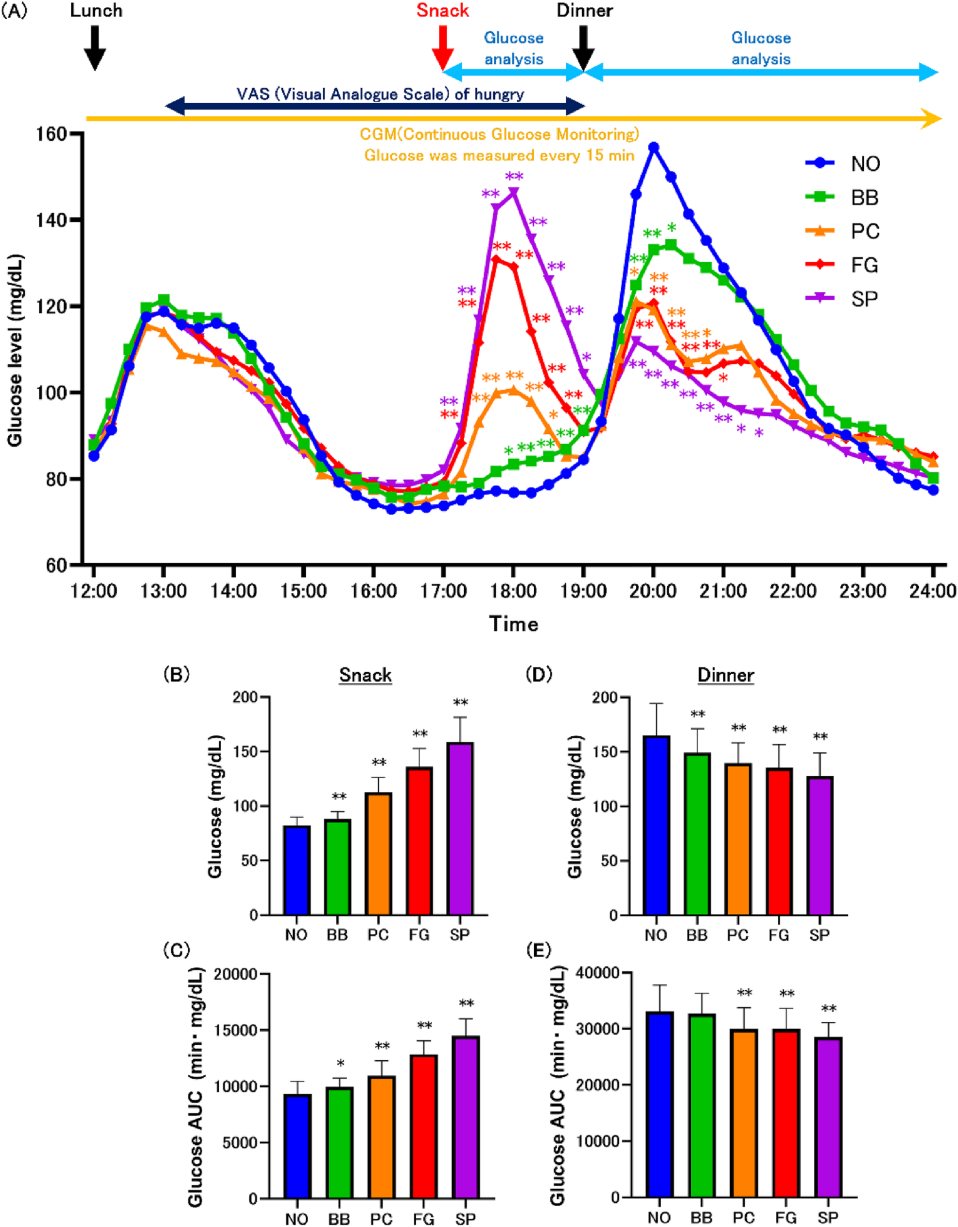
Response of glucose levels at 17:00 snack. Monitoring of glucose levels at 17:00 snack (**A**). Maximum glucose after snack intake (**B**). Area under the curve (AUC) of glucose levels after snack intake (**C**). Maximum glucose after dinner intake (**D**). AUC of glucose levels after dinner intake (**E**). Values are means ± standard deviations. Statistics vs. NO group, *p < 0.05, **p < 0.01. *NO* no-snack, *BB* fried black beans snack, *PC* potato chips snack, *FG* fruit granola snack, *SP* roasted sweet potato snack

At first, when we examined the changes in glucose levels during the snack at 17:00, the postprandial glucose levels in the BB, PC, FG, and SP groups were elevated (Fig. 3A, Supplemental Table S6). Compared with the change in maximal glucose level during the snack, BB, PC, FG, and SP groups showed increases in maximal glucose levels compared with NO group (Fig. 3B, Supplemental Table S7). The AUC similarly showed increases in the BB, PC, FG, and SP groups (Fig. 3C, Supplemental Table S7).

Next, when analyzing the glucose level variability at dinner, the pre-dinner glucose levels in BB and SP groups were higher than those in NO group at 19:00 (Fig. 3A, Supplemental Table S6). The postprandial glucose level in BB group was lower than that in NO group at 19:45, 20:00, and 20:15. The postprandial glucose level in the PC group was lower than that in the NO group at 19:45, 20:00, 20:15, 20:30, and 20:45. The postprandial glucose level in the FG group was lower than that in the NO group at 19:45, 20:00, 20:15, 20:30, 20:45, and 21:00. The postprandial glucose level in the SP group was lower than that in the NO group at 19:45, 20:00, 20:15, 20:30, 20:45, 21:00, 21:15, and 21:30. Compared with the change in the maximal glucose level during the dinner, BB, PC, FG, and SP groups showed suppressed increases in the maximal glucose level compared with NO group (Fig. 3D, Supplemental Table S7). The AUC after dinner similarly showed a suppressed increase in the PC, FG, and SP groups (Fig. 3E, Supplemental Table S7).

### Total AUC and Correlation between snacking and dinner

**Fig. 4:**
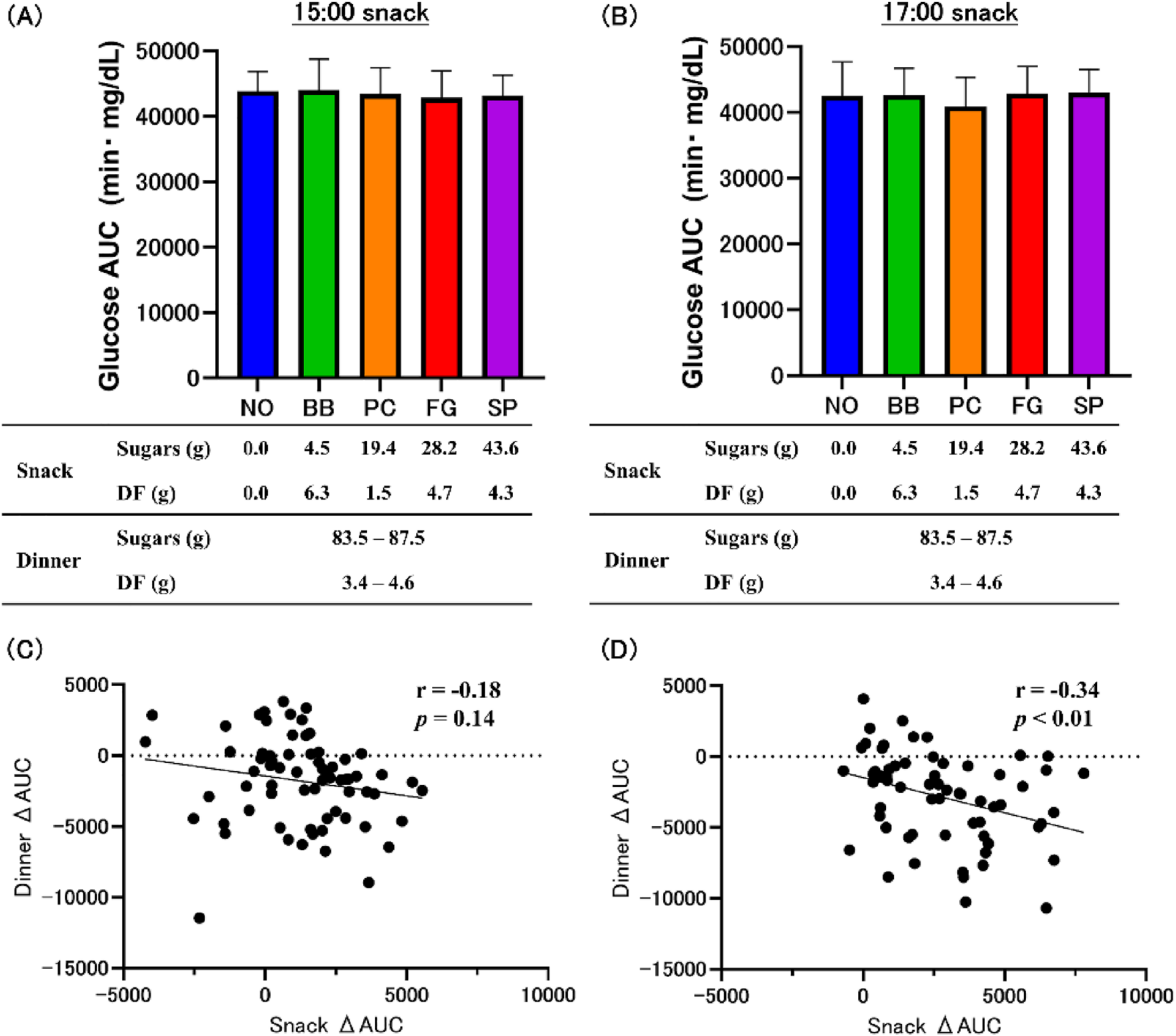
Analysis of total AUC. Total AUC at 15:00 snacking (**A**) and 17:00 snacking (**B**). Values are means ± standard deviations. Statistics vs. NO group. Correlation coefficient for snack AUC and dinner AUC at 15:00 snack (**C**) and 17:00 snack (**D**). *NO* no-snack, *BB* fried black beans snack, *PC* potato chips snack, *FG* fruit granola snack, *SP* roasted sweet potato snack, *AUC* area under the curve, *DF* dietary fiber

The amount of sugars a subject consumed increased with snacking: BB, 4.5 g; PC, 19.4 g; FG, 28.2 g; and SP, 43.6 g. Snacking at 15:00 did not show a significant difference in the total AUC (Fig. 4A, Supplemental Table S8). Moreover, snacking at 17:00 did not show a significant difference in the total AUC (Fig. 4B, Supplemental Table S8).

When the correlations were analyzed for the AUC of snacks and AUC of dinner, no correlation was found for the 15:00 snack (Fig. 4C). On the other hand, a negative correlation between the snack AUC and dinner AUC was observed for snacks at 17:00 (Fig. 4D).

### Amplitude range of glucose and distribution of glucose

**Fig. 5:**
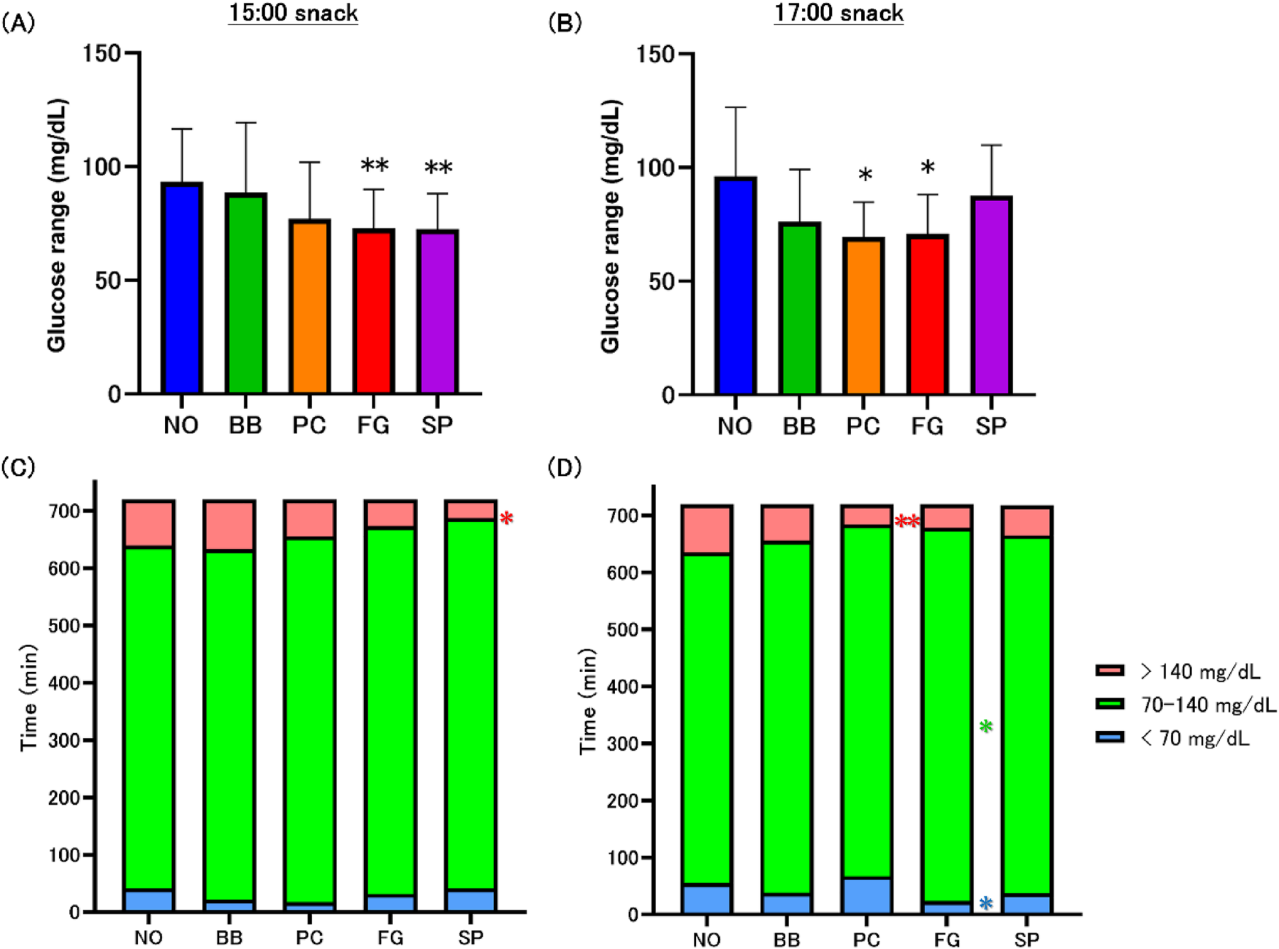
Analysis of amplitude range of glucose and distribution of glucose. The amplitude range of the glucose was defined as the difference between the maximum and minimum glucose during glucose monitoring at 15:00 snacking (**A**) and 17:00 snacking (**B**). Values are means ± standard deviations. The time of distribution of glucose was classified as < 70, 70–140and > 140 mg/dL for 15:00 snacking (**C**) and 17:00 snacking (**D**). Values of time (min) are means. Statistics vs. NO group, *p < 0.05, **p < 0.01. *NO* no-snack, *BB* fried black beans snack, *PC* potato chips snack, *FG* fruit granola snack, *SP* roasted sweet potato snack

To observe the range of glucose variability, the amplitude range of glucose was defined as the difference between the maximum and minimum glucose during glucose monitoring. The glucose range in the FG and SP groups was significantly lower than that in the NO group at the 15:00 snack (Fig. 5A, Supplemental Table S9). At the 17:00 snack, the glucose range in the PC and FG groups was significantly lower than that in the NO group (Fig. 5B, Supplemental Table S9).

The glucose levels were categorized every 15 min from 12:00 to 24:00 into three groups— < 70 mg/dL (hypoglycemic), 70–140 mg/dL (appropriate ranges), and > 140 mg/dL or higher (hyperglycemic) > and the time of each group during glucose monitoring was calculated [29–31]. For snacking at 15:00, the distribution in hyperglycemia in the SP group was significantly lower than that in the NO group (Fig. 5C, Supplemental Table S10). For snacking at 17:00, the distribution in hypoglycemia in the FG group was significantly lower than that in the NO group (Fig. 5D, Supplemental Table S11). The distribution in the 70–140 mg/dL range in the FG group was significantly higher than that in the NO group. The distribution in hyperglycemia in the PC group was significantly lower than that in the NO group.

### Correlation between AUC and nutrients

**Fig. 6:**
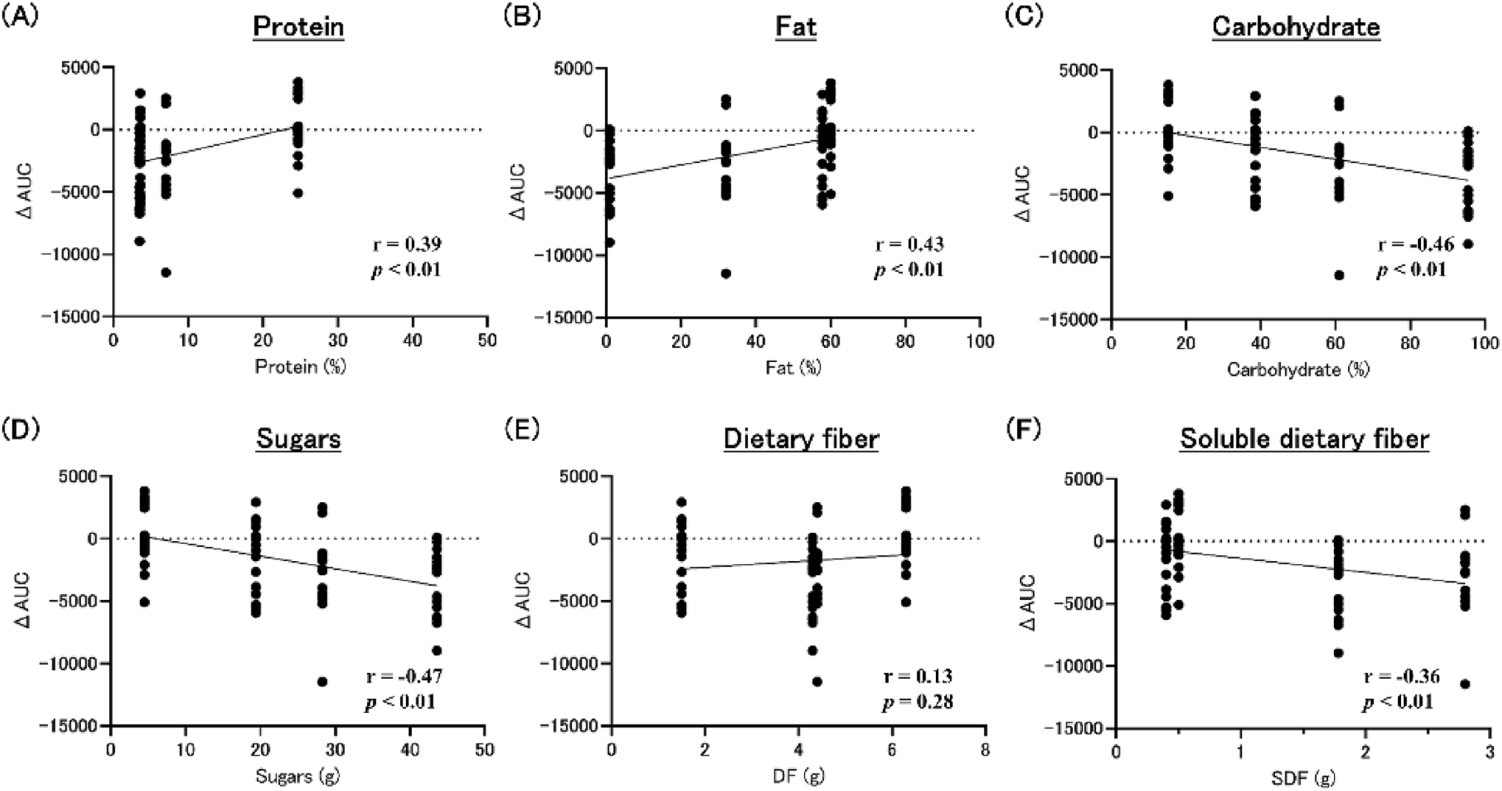
Correlation coefficient for nutrients at 15:00 snack. *DF* dietary fiber, *SDF* soluble dietary fiber, *AUC* area under the curve

The correlation between the nutrients of the snacks and AUC at dinner was analyzed. Protein and fat showed a positive correlation, while carbohydrate showed a negative correlation for the 15:00 snack (Fig. 6A–C). To analyze carbohydrates, we also looked for correlations between sugars and dietary fiber. Sugars showed a negative correlation (Fig. 6D). Dietary fiber showed no correlation (r = 0.13, *p* = 0.28, Fig. 6E). Among dietary fibers, soluble dietary fiber showed a negative correlation (Fig. 6F).

**Fig. 7:**
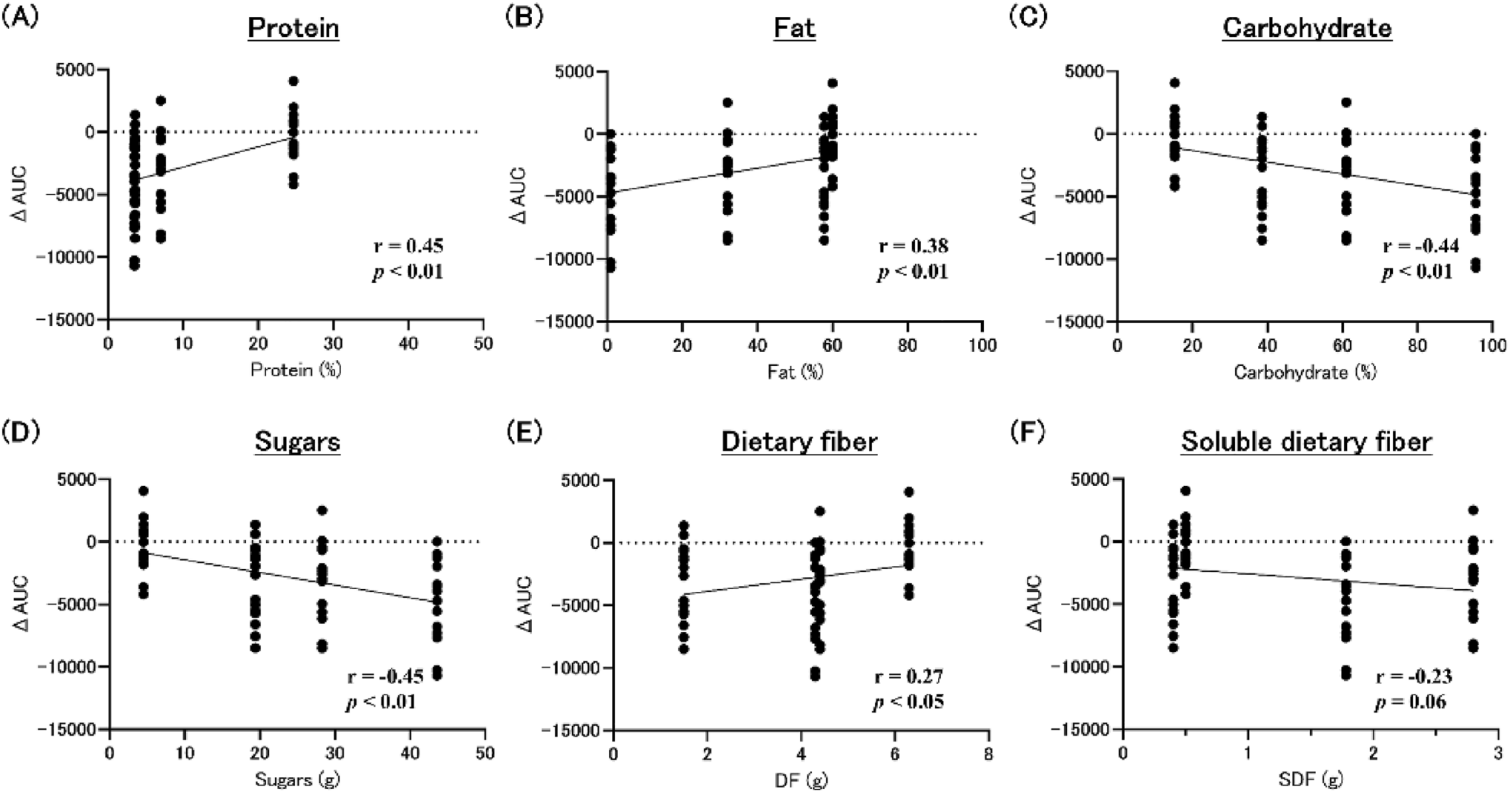
Correlation coefficient for nutrients at 17:00 snack. *DF* dietary fiber, *SDF* soluble dietary fiber, *AUC* area under the curve

In the 17:00 snacking groups, protein and fat showed a positive correlation, while carbohydrate showed a negative correlation (Fig. 7A–C). Sugars showed a negative correlation (Fig. 7D). Dietary fiber showed a positive correlation (r = 0.27, *p* < 0.05, Fig. 7E). Soluble dietary fiber showed a trend to negative correlation (Fig. 7F).

### VAS of hunger

In the 15:00 snacking groups, when analyzing the VAS of hunger until dinner, the BB, PC, FG, and SP groups showed lower values than NO group between 16:00 and 17:00 (Supplemental Figure S3A). The values for BB, PC, and SP groups were lower than those for NO group at 18:00, while those for BB, PC, and FG groups were lower than those for NO group at 19:00.

In the 17:00 snacking groups, when analyzing the VAS of hunger until dinner, the values for BB, PC, FG, and SP groups were lower than those for NO group at 18:00 (Supplemental Figure. S3B). Those for BB, PC, and SP groups were lower than those for NO group at 19:00.

## Discussion

In this study, we found that (1) eating a 838 kJ (= 200 kcal) snack two hours before dinner suppressed the increase in glucose levels after dinner; (2) eating a 838 kJ roasted sweet potato and fruit granola as a snack four hours before dinner suppressed the rise in glucose after dinner; (3) snacking on 838 kJ of fried black beans, potato chips, fruit granola, and roasted sweet potato did not increase the total AUC (snack and dinner) compared with no snacking, despite the increased amount of carbohydrates; and (4) consuming snacks decreased the duration in which glucose levels were hyperglycemic and hypoglycemic.

### Effect of fasting time

It has been reported that the longer the fasting time, the higher the levels of postprandial blood glucose [10]. Compared with the no-snack group, the four hours of fasting for the 15:00 snack group and two hours of fasting for the 17:00 snack group may have caused the NO group, which had fasted for seven hours, to be more susceptible to having elevated glucose levels after dinner.

### Effects of a black bean snack

Black soybeans are a low-GI food, as reported in previous studies [33], and the sugar intake resulting from the consumption of black bean snacks in this study was low at 4.5 g (Table 2). The increase in blood glucose levels after snacking on black bean snacks was, therefore, also low, since only 4.5 g of sugar was ingested of characteristically low-GI food. (Figs. 2A–C, 3A–C). Eating a high-protein breakfast is reported to reduce feelings of hunger throughout the day [34]. Eating high-protein soy snacks between meals reportedly keeps people feeling full until dinner [35]. Although the black bean snack (BB) group received protein-rich snacks, there was no difference in satiety compared with the groups that received other snacks, suggesting that the protein content had little effect. A second-meal effect resulting from consumption of black soybeans was observed; we considered the reason for this. Of the prepared snacks under investigation, only black soybeans contain high amounts of protein (12.3 g). In a study examining the second-meal effect on pre-diabetic subjects consuming 18 g of soybean powder, it was proposed that the test food should be consumed 30 min before the meal [36]. In our recent papers, we demonstrated that a high-protein breakfast or lunch results in a second-meal effect on dinner time [37, 38]. Although the mechanism of protein-rich food on the second-meal effect remains unknown, consumption of a protein-rich snack—similarly to consumption of a protein-rich breakfast and lunch—before dinner may help in reducing the rapid postprandial increase in blood glucose. Consumption of the 38.5 g BB in this study induced the second-meal effect in 2 h rather than 4 h, suggesting that the relationship between time of snack intake and energy intake, including lunch, is an important factor when considering the effects of snacking on black soybeans.

### Effects of a potato chip snack

Potato chips produced a second-meal effect by suppressing the increase in the blood glucose level at dinner in the 17:00 snack group (Fig. 3A, D, E). Moreover, the GI of potato chips is around 60, and another study reported that its AUC is significantly lower than that of crackers [13, 39]. In the 17:00 snack of the PC group, consumption resulted in a lower glucose amplitude and shorter time spent in hyperglycemia (Fig. 5B, D). Potato chips may also be less likely to adversely affect blood glucose levels if consumed at an appropriate time. The black bean and potato chip snacks used in the current experiments contain high levels of fat. Therefore, in future experiments, we should examine other aspects related to the healthiness of snacks; for example, longer periods during which there is intake of different fat types, such as omega-3, -6, and -9 fatty acids.

### Effects of fruit granola and sweet potato snacks

In the fruit granola snack (FG) and sweet potato snack (SP) groups, there was a suppression of the maximum increase in blood glucose after dinner in both the 15:00 and 17:00 snack groups, with the AUCs being lower than those of the NO group (Figs. 2A, D, E and 3A, D, E). It has been reported that the glycemic index of granola and muesli is 56 (range 39–70) [22]. GIs of 80 for roasted sweet potatoes and 55 for raw sweet potatoes have been reported [16, 17]. Grains that reduce the rise in blood sugar include foods high in dietary fiber, such as barley and oats. There are two types of dietary fiber: insoluble fiber and soluble fiber. Soluble fiber is highly viscous and has been reported to slow down the rate of food movement from the stomach to the small intestine and the digestion of carbohydrates in the small intestine, resulting in a gradual increase in blood sugar levels [40]. Fruit granola and roasted sweet potatoes contain 4.4 and 4.3 g of dietary fiber, respectively (Table 2). They also contain 2.8 and 1.8 g of soluble fiber, respectively. Oats, the main ingredient in fruit granola, contain a specific soluble fiber, β-glucan. It has been reported that the β-glucan in oats has a moderating effect on glucose absorption, and it is possible that the fruit granola group experienced a slower increase in postprandial blood glucose levels due to the intake of dietary fiber [41]. There are two types of effects resulting from consumption of soluble dietary fibers such as β-glucan: the direct inhibition of glucose absorption through viscosity and indirect inhibition of blood glucose elevation through bacteria [42, 43]. Intestinal bacteria in the large intestine proliferate based on soluble dietary fiber and produce short-chain fatty acids, such as butyric acid and acetic acid [43]. Short-chain fatty acids (SCFAs) stimulate L-cells in the small intestine and gut to secrete the hormone glucagon-like peptide (GLP)-1 [44]. GLP-1 enhances insulin function, and sustained insulin secretion before dinner is also thought to have a second-meal effect that suppresses postprandial blood glucose elevation after dinner [45]. In addition, it has been reported that oat β-glucan leads to the elevation of total SCFAs and acetate after four hours of reaction in a model of intestinal fermentation [46]. On the other hand, roasted sweet potatoes have also been reported to improve intestinal microflora in healthy Japanese women consuming 100 and 300 g of roasted sweet potatoes [47]. It has also been reported that the oral administration of sweet potato leaf powder suppresses postprandial hyperglycemia through the promotion of GLP-1 secretion [48]. In the present study, there was also a negative correlation between the AUC at dinner and soluble fiber (Fig. 6F). Furthermore, the fact that no second-meal effect was observed with the glucose snack at 15:00 in the optional study (Supplemental Figure S2A) suggests that soluble dietary fiber may be an important factor in achieving a second-meal effect in the 15:00 snack group. The mechanism of action may be the direct inhibition of glucose absorption in the intestinal tract and indirect enhancement of insulin action via GLP-1. However, since the secretion of hormones such as insulin and GLP-1 is important for these effects, it is important to employ hormone monitoring in future studies.

### Second-meal effect and dietary fiber

In our recent experiments on dietary fiber-rich snacking between lunch and dinner, we observed a second-meal effect at dinner [9, 10]. The findings indicated that eating biscuit snacks alongside mulberry or barley leaves 4 h before dinner was an effective way to suppress postprandial glucose levels after dinner in young adults who prefer eating in the evenings [9]. In cross-over snacking intervention experiments, the increases in glucose at dinner time 3 h after snacking were significantly lower in the group that snacked on fiber-rich biscuits containing 18.4 g carbohydrate (9.2 g starch/sugar and 9.2 g dietary fiber) than in the group that ate low-fiber snacks (18.5 g: 18.1 g starch/sugar and 0.4 g dietary fiber) [10]. These studies have suggested the importance of dietary fibers in determining the second-meal effect, although the involved mechanisms have not yet been clearly elucidated. Dietary fiber affects the composition and metabolic activity of the microbiome and levels of short-chain fatty acids [49]. However, we do not know whether the period of 2–4 h after dietary fiber-rich snacking is long enough to affect microbiota in the small and large intestines. Notably, the microbiome can be quickly and profoundly altered by common human actions and experiences [50]. For example, a change in host fiber intake is positively correlated with a change of 15% in gut microbiota abundance on the next day [50]. A study of 24 h food records and with fecal shotgun metagenomic analysis of 34 healthy humans collected daily over a 17-day period suggested that daily fluctuations in the microbiome are associated with food choices [51]. As similar foods have different effects on the microbiome of different people [51], it will be necessary in the future to examine the effects of fruit granola and sweet potato as dietary fiber-rich foods on the microbiome of individual subjects.

### Glucose control

When the blood glucose level falls below 70 mg/dL, hypoglycemic symptoms may appear [52]. On the other hand, a blood glucose level above 140 mg/dL is referred to as a blood glucose spike, and hyperglycemia is known to injure the vascular endothelium and increase the risk of diabetes and cardiac events [3]. In 2019, the concept of time in range (TIR), a glycemic control index, was also presented for diabetic patients [53]. Repetitive glucose spikes promote atherosclerotic lesion formation in C57BL/6 mice [54, 55]. In other words, the concept of controlling blood glucose levels within a certain range is supported. In this study, we suggest the advantages of PC, FG, and SP as snacks for controlling blood glucose stability based on the amplitude range and distribution of glucose. The correlation between snack nutrients and AUC at dinner was analyzed, and carbohydrate and soluble dietary fiber showed a negative correlation. Moderate intake of carbohydrates and soluble fiber at appropriate times may be important for controlling blood glucose at dinner.

## Conclusion

In this study, the intake of carbohydrates and soluble fiber through snacks was an important factor influencing the second-meal effect at dinner. Consuming snacks stabilizes the changes associate with hyperglycemic and hypoglycemic events. These results may contribute to the development of snacking research and also second-meal effect research. As a future study, we would like to examine similar effects and optimal times of the day to snack for those who require stricter blood glucose control. Regarding other effects, we would like to examine changes in intestinal flora and the intake of snacks high in soluble fiber, such as fruit granola and sweet potatoes.

## Supplementary Information

Below is the link to the electronic supplementary material.

Supplementary file1 (DOCX 1507 KB)

## Data Availability

Secondary use for commercial purposes is also permitted under the main condition that the original author's credit (name, title of work, etc.) is displayed.

## References

[1] McClain DA (2002). Hexosamines as mediators of nutrient sensing and regulation in diabetes. J Diabetes Complications.

[2] Parkin CG, Buskirk A, Hinnen DA, Axel-Schweitzer M (2012). Results that matter: structured vs. unstructured self-monitoring of blood glucose in type 2 diabetes. Diabetes Res Clin Pract.

[3] Brand-Miller J, Dickinson S, Barclay A, Celermajer D (2007). The glycemic index and cardiovascular disease risk. Curr Atheroscler Rep.

[4] Kajiyama S, Imai S, Hashimoto Y, Yamane C, Miyawaki T, Matsumoto S, Ozasa N, Tanaka M, Kajiyama S, Fukui M (2018). Divided consumption of late-night-dinner improves glucose excursions in young healthy women: a randomized cross-over clinical trial. Diabetes Res Clin Pract.

[5] Imai S, Kajiyama S, Hashimoto Y, Nitta A, Miyawaki T, Matsumoto S, Ozasa N, Tanaka M, Kajiyama S, Fukui M (2018). Consuming snacks mid-afternoon compared with just after lunch improves mean amplitude of glycaemic excursions in patients with type 2 diabetes: A randomized crossover clinical trial. Diabetes Metab.

[6] Jenkins DJ, Wolever TM, Taylor RH, Griffiths C, Krzeminska K, Lawrie JA, Bennett CM, Goff DV, Sarson DL, Bloom SR (1982). Slow release dietary carbohydrate improves second meal tolerance. Am J Clin Nutr.

[7] Wolever TM, Jenkins DJ, Ocana AM, Rao VA, Collier GR (1988). Second-meal effect: low-glycemic-index foods eaten at dinner improve subsequent breakfast glycemic response. Am J Clin Nutr.

[8] Nitta A, Imai S, Kajiyama S, Miyawaki T, Matsumoto S, Ozasa N, Kajiyama S, Hashimoto Y, Tanaka M, Fukui M (2019). Impact of different timing of consuming sweet snack on postprandial glucose excursions in healthy women. Diabetes Metab.

[9] Kuwahara M, Kim HK, Ozaki M, Nanba T, Chijiki H, Fukazawa M, Okubo J, Mineshita Y, Takahashi M, Shibata S (2020). Consumption of biscuits with a beverage of mulberry or barley leaves in the afternoon prevents dinner-induced high, but not low, increases in blood glucose among young adults. Nutrients.

[10] Kim HK, Nanba T, Ozaki M, Chijiki H, Takahashi M, Fukazawa M, Okubo J, Shibata S (2020). Effect of the intake of a snack containing dietary fiber on postprandial glucose levels. Foods.

[11] Masutomi H, Ishihara K, Hirao K, Furutani A (2021). Taking fruit granola as a snacks can affect post-dinner glucose levels and sleep quality. J Food Nutr.

[12] Reyniers S, Ooms N, Delcour JA (2020). Transformations and functional role of starch during potato crisp making: a review. J Food Sci.

[13] Brand JC, Nicholson PL, Thorburn AW, Truswell AS (1985). Food processing and the glycemic index. Am J Clin Nutr.

[14] Lai YC, Huang CL, Chan CF, Lien CY, Liao WC (2013). Studies of sugar composition and starch morphology of baked sweet potatoes (*Ipomoea batatas* (L.) Lam). J Food Sci Technol.

[15] Chan CF, Chiang CM, Lai YC, Huang CL, Kao SC, Liao WC (2014). Changes in sugar composition during baking and their effects on sensory attributes of baked sweet potatoes. J Food Sci Technol.

[16] Chen YY, Lai MH, Hung HY, Liu JF (2013). Sweet potato [*Ipomoea batatas* (L.) Lam. “Tainong 57”] starch improves insulin sensitivity in high-fructose diet-fed rats by ameliorating adipocytokine levels, pro-inflammatory status, and insulin signaling. J Nutr Sci Vitaminol (Tokyo).

[17] Bahado-Singh PS, Wheatley AO, Ahmad MH, Morrison EY, Asemota HN (2006). Food processing methods influence the glycaemic indices of some commonly eaten West Indian carbohydrate-rich foods. Br J Nutr.

[18] Ooi CP, Loke SC (2013). Sweet potato for type 2 diabetes mellitus. Cochrane Database Syst Rev.

[19] Sievenpiper JL, Kendall CW, Esfahani A, Wong JM, Carleton AJ, Jiang HY, Bazinet RP, Vidgen E, Jenkins DJ (2009). Effect of non-oil-seed pulses on glycaemic control: a systematic review and meta-analysis of randomised controlled experimental trials in people with and without diabetes. Diabetologia.

[20] Escobedo A, Rivera-Leon EA, Luevano-Contreras C, Urias-Silvas JE, Luna-Vital DA, Morales-Hernandez N, Mojica L (2021). Common bean baked snack consumption reduces apolipoprotein B-100 levels: a randomized crossover trial. Nutrients.

[21] Sanchez-Tapia M, Hernandez-Velazquez I, Pichardo-Ontiveros E, Granados-Portillo O, Galvez A, Armando RT, Torres N (2020). Consumption of cooked black beans stimulates a cluster of some clostridia class bacteria decreasing inflammatory response and improving insulin sensitivity. Nutrients.

[22] Tosh SM, Chu Y (2015). Systematic review of the effect of processing of whole-grain oat cereals on glycaemic response. Br J Nutr.

[23] Mather K, Boachie R, Anini Y, Panahi S, Anderson GH, Luhovyy BL (2020). Effects of cultured dairy and nondairy products added to breakfast cereals on blood glucose control, satiation, satiety, and short-term food intake in young women. Appl Physiol Nutr Metab.

[24] Ministry of Health, Labor and Welfare. https://www.e-healthnet.mhlw.go.jp/information/food/e-03-013.html. Accessed 6 Feb 2023

[25] Hoss U, Budiman ES (2017). Factory-calibrated continuous glucose sensors: the science behind the technology. Diabetes Technol Ther.

[26] Yamawaki T, Hamada N, Zhang J, Yamaguchi E, Komuro H (2019). Glucose profiles analysis using the Free Style Libre Pro^®^ in 3 cases of total gastrectomy without hypoglycemic symptoms. J Jpn Assoc Rural Med.

[27] Kumagai R, Muramatsu A, Fujii M, Katakura Y, Ito K, Fujie K, Nakata Y, Hashimoto K, Yagyu H (2019). Comparison of glucose monitoring between Freestyle Libre Pro and iPro2 in patients with diabetes mellitus. J Diabetes Investig.

[28] Bailey T, Bode BW, Christiansen MP, Klaff LJ, Alva S (2015). The performance and usability of a factory-calibrated flash glucose monitoring system. Diabetes Technol Ther.

[29] Zhong VW, Crandell JL, Shay CM, Gordon-Larsen P, Cole SR, Juhaeri J, Kahkoska AR, Maahs DM, Seid M, Forlenza GP, Mayer-Davis EJ (2017). Dietary intake and risk of non-severe hypoglycemia in adolescents with type 1 diabetes. J Diabetes Complicat.

[30] International Diabetes Federation Guideline Development Group (2014). Guideline for management of postmeal glucose in diabetes. Diabetes Res Clin Pract.

[31] Yagi M, Yonei Y (2019). Glycative stress and anti-aging: 13. Regulation of glycative stress. 1. Postprandial blood glucose regulation. Glycative Stress Res.

[32] Kung B, Anderson GH, Pare S, Tucker AJ, Vien S, Wright AJ, Goff HD (2018). Effect of milk protein intake and casein-to-whey ratio in breakfast meals on postprandial glucose, satiety ratings, and subsequent meal intake. J Dairy Sci.

[33] Winham DM, Hutchins AM, Thompson SV (2017). Glycemic response to black beans and chickpeas as part of a rice meal: a randomized cross-over trial. Nutrients.

[34] Leidy HJ, Lepping RJ, Savage CR, Harris CT (2011). Neural responses to visual food stimuli after a normal vs. higher protein breakfast in breakfast-skipping teens: a pilot fMRI study. Obesity.

[35] Leidy HJ, Todd CB, Zino AZ, Immel JE, Mukherjea R, Shafer RS, Ortinau LC, Braun M (2015). Consuming high-protein soy snacks affects appetite control, satiety, and diet quality in young people and influences select aspects of mood and cognition. J Nutr.

[36] Liu Y, Jiang H, Ruan B, Liu Y, Le S, Fu X, Wang S (2022). Effect of high-protein vs. high-fat snacks before lunch on glycemic variability in prediabetes: a study protocol for a randomized controlled trial. Front Nutr.

[37] Xiao K, Furutani A, Sasaki H, Takahashi M, Shibata S (2023). Effect of a high protein diet at breakfast on postprandial glucose level at dinner time in healthy adults. Nutrients.

[38] Kuwahara M, Kim HK, Furutani A, Mineshita Y, Nakaoka T, Shibata S (2022). Effect of lunch with different calorie and nutrient balances on dinner-induced postprandial glucose variability. Nutr Metab (Lond).

[39] Moser S, Aragon I, Furrer A, Van Klinken JW, Kaczmarczyk M, Lee BH, George J, Hamaker BR, Mattes R, Ferruzzi MG (2018). Potato phenolics impact starch digestion and glucose transport in model systems but translation to phenolic rich potato chips results in only modest modification of glycemic response in humans. Nutr Res.

[40] Tabatabai A, Li S (2000). Dietary fiber and type 2 diabetes. Clin Excell Nurse Pract.

[41] Granfeldt Y, Nyberg L, Bjorck I (2008). Muesli with 4 g oat beta-glucans lowers glucose and insulin responses after a bread meal in healthy subjects. Eur J Clin Nutr.

[42] Angelov A, Yaneva-Marinova T, Gotcheva V (2018). Oats as a matrix of choice for developing fermented functional beverages. J Food Sci Technol.

[43] Miyamoto J, Watanabe K, Taira S, Kasubuchi M, Li X, Irie J, Itoh H, Kimura I (2018). Barley beta-glucan improves metabolic condition via short-chain fatty acids produced by gut microbial fermentation in high fat diet fed mice. PLoS One.

[44] Hernandez MAG, Canfora EE, Jocken JWE, Blaak EE (2019). The short-chain fatty acid acetate in body weight control and insulin sensitivity. Nutrients.

[45] Johansson EV, Nilsson AC, Ostman EM, Bjorck IM (2013). Effects of indigestible carbohydrates in barley on glucose metabolism, appetite and voluntary food intake over 16 h in healthy adults. Nutr J.

[46] Queenan KM, Stewart ML, Smith KN, Thomas W, Fulcher RG, Slavin JL (2007). Concentrated oat beta-glucan, a fermentable fiber, lowers serum cholesterol in hypercholesterolemic adults in a randomized controlled trial. Nutr J.

[47] Banno T, Komori Y, Suzuki S, Tanabe K, Kasaoka S, Benno Y (2016). Sweet potato improves defecation and gut microbiota of female university students. Nippon Eiyo Shokuryo Gakkaishi.

[48] Nagamine R, Ueno S, Tsubata M, Yamaguchi K, Takagaki K, Hira T, Hara H, Tsuda T (2014). Dietary sweet potato (*Ipomoea batatas* L.) leaf extract attenuates hyperglycaemia by enhancing the secretion of glucagon-like peptide-1 (GLP-1). Food Funct.

[49] Simpson HL, Campbell BJ (2015). Review article: dietary fibre–microbiota interactions. Aliment Pharmacol Ther.

[50] Lawrence AD, Arne CM, Jonathan F, Maria IC-B, Matthew CB, Allison P, Susan EE, Eric JA (2014). Host lifestyle affects human microbiota on daily timescales. Genome Biol.

[51] Abigail JJ, Pajau V, Gabriel AAl-G, Benjamin MH, Tonya LW, Robin RS-C, Austin DK, Anna KS, Arzang NS, Personalized Microbiome Class Students; Jens W, Ravi M, Katie K, Dan K (2019) Daily sampling reveals personalized diet-microbiome associations in humans. Cell Host Microbe 25(6):789-802.e5. doi: 10.1016/j.chom.2019.05.005 10.1016/j.chom.2019.05.005 31194939

[52] Shah VN, DuBose SN, Li Z, Beck RW, Peters AL, Weinstock RS, Kruger D, Tansey M, Sparling D, Woerner S, Vendrame F, Bergenstal R, Tamborlane WV, Watson SE, Sherr J (2019). Continuous glucose monitoring profiles in healthy nondiabetic participants: a multicenter prospective study. J Clin Endocrinol Metab.

[53] Battelino T, Danne T, Bergenstal RM, Amiel SA, Beck R, Biester T, Bosi E, Buckingham BA, Cefalu WT, Close KL, Cobelli C, Dassau E, DeVries JH, Donaghue KC, Dovc K, Doyle FJ, Garg S, Grunberger G, Heller S, Heinemann L, Hirsch IB, Hovorka R, Jia W, Kordonouri O, Kovatchev B, Kowalski A, Laffel L, Levine B, Mayorov A, Mathieu C, Murphy HR, Nimri R, Norgaard K, Parkin CG, Renard E, Rodbard D, Saboo B, Schatz D, Stoner K, Urakami T, Weinzimer SA, Phillip M (2019). Clinical targets for continuous glucose monitoring data interpretation: recommendations from the international consensus on time in range. Diabetes Care.

[54] Shuto Y, Asai A, Nagao M, Sugihara H, Oikawa S (2015). Repetitive glucose spikes accelerate atherosclerotic lesion formation in C57BL/6 mice. PLoS One.

[55] Rogers SC, Zhang X, Azhar G, Luo S, Wei JY (2013). Exposure to high or low glucose levels accelerates the appearance of markers of endothelial cell senescence and induces dysregulation of nitric oxide synthase. J Gerontol A Biol Sci Med Sci.

