# Supplementary material for: Effects of intake of four types of snack with different timings on postprandial glucose levels after dinner
Source: Eur J Nutr. 2023 Apr 15;62(5):2217–31. doi: 10.1007/s00394-023-03138-4 (PMC10349787; doi:10.1007/s00394-023-03138-4)
Supplement: Supplementary file 1 — Supplementary file1 (DOCX 1507 KB) [file 394_2023_3138_MOESM1_ESM.docx]

**Supplemental dates**

**
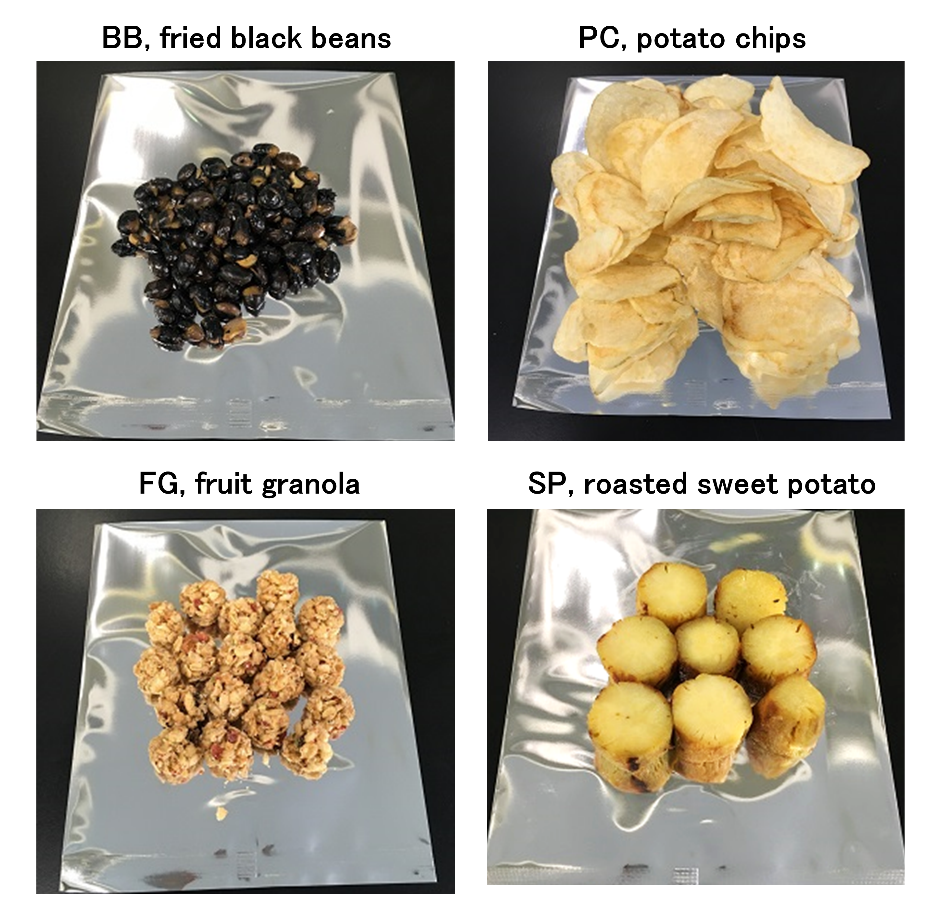
**

**Supplemental Fig. S1. Test meal of snack**

BB, fried black beans snack; PC, potato chips snack; FG, fruit granola snack; SP, roasted sweet potato snack. All four snacks had consistent calorie counts of 838 kJ.


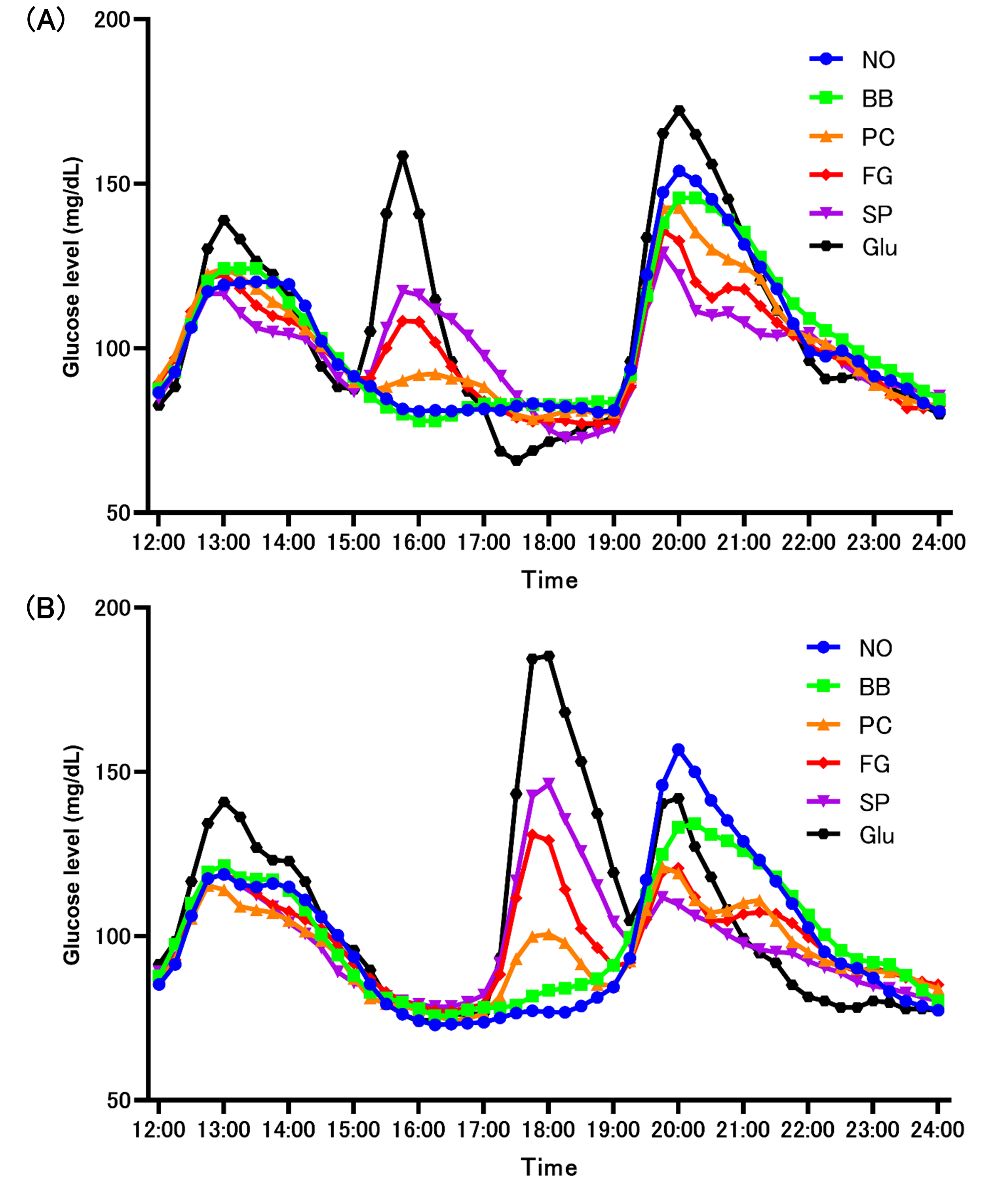


**Supplemental Fig. S2. Response of glucose levels.**

Monitoring of glucose levels at 15:00 snack (A) and 17:00 snack (B). Values are means. NO, no-snack; BB, fried black beans snack; PC, potato chips snack; FG, fruit granola snack; SP, roasted sweet potato snack; Glu, glucose snack.

**
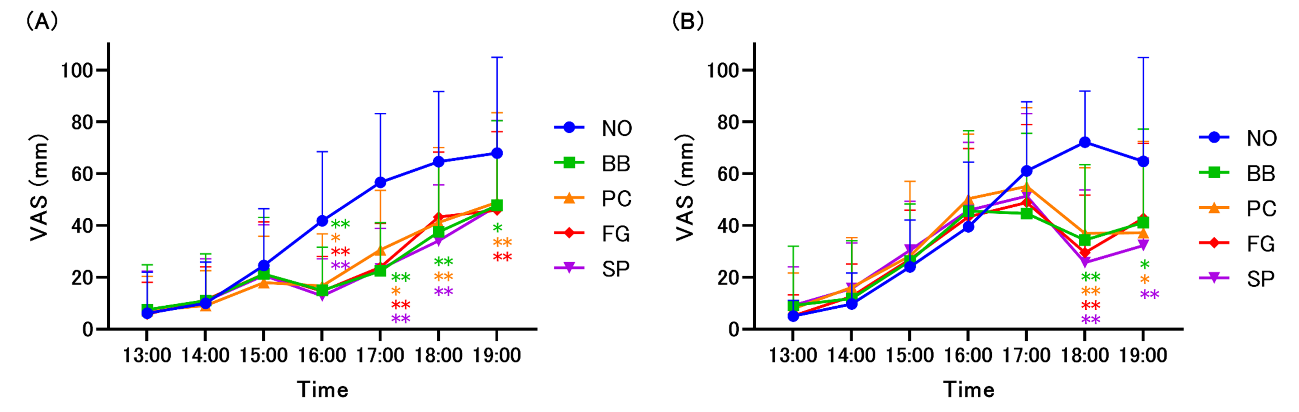
**

**Supplemental Fig. S3. VAS of hunger.**

Feeling hunger until dinner at 15:00 snack (A). Feeling hunger until dinner at 15:00 snack (B). Values are means ± standard deviation. Two way-repeated-ANOVA was used for VAS and Dunnett's was used for multiple testing vs NO group, * *p* < 0.05, ** *p* < 0.01. NO, no-snack; BB, fried black beans snack; PC, potato chips snack; FG, fruit granola snack; SP, roasted sweet potato snack.

**Supplemental Table S1. Randomized of test schedule (lunch, snack, and dinner contents).**

| Subject No | Day 1 | Day 2 | Day 3 | Day4 | Day 5 |
| --- | --- | --- | --- | --- | --- |
| 15-1  / 17-1 | Hashed beef  **Sweet potato**  Hamburger steak | Butter chicken curry  **Potato chips**  Cheese-in-hamburger steak | Beef curry  **No snack**  Fried chicken | Cream stew  **Black bean**  Hamburger steak | Hashed beef  **Fruit granola**  Cheese-in-hamburger steak |
| 15-2  / 17-2 | Hashed beef  **Potato chips**  Hamburger steak | Butter chicken curry  **No snack**  Cheese-in-hamburger steak | Beef curry  **Black bean**  Fried chicken | Cream stew  **Fruit granola**  Hamburger steak | Hashed beef  **Sweet potato**  Cheese-in-hamburger steak |
| 15-3  / 17-3 | Hashed beef  **No snack**  Hamburger steak | Butter chicken curry  **Black bean**  Cheese-in-hamburger steak | Beef curry  **Fruit granola**  Fried chicken | Cream stew  **Sweet potato**  Hamburger steak | Hashed beef  **Potato chips**  Cheese-in-hamburger steak |
| 15-4  / 17-4 | Hashed beef  **Black bean**  Hamburger steak | Butter chicken curry  **Fruit granola**  Cheese-in-hamburger steak | Beef curry  **Sweet potato**  Fried chicken | Cream stew  **Potato chips**  Hamburger steak | Hashed beef  **No snack**  Cheese-in-hamburger steak |
| 15-5  / 17-5 | Hashed beef  **Fruit granola**  Hamburger steak | Butter chicken curry  **Sweet potato**  Cheese-in-hamburger steak | Beef curry  **Potato chips**  Fried chicken | Cream stew  **No snack**  Hamburger steak | Hashed beef  **Black bean**  Cheese-in-hamburger steak |
| 15-6  / 17-6 | Butter chicken curry  **Sweet potato**  Fried chicken | Beef curry  **Potato chips**  Hamburger steak | Cream stew  **No snack**  Cheese-in-hamburger steak | Hashed beef  **Black bean**  Fried chicken | Beef curry  **Fruit granola**  Hamburger steak |
| 15-7  / 17-7 | Butter chicken curry  **Potato chips**  Fried chicken | Beef curry  **No snack**  Hamburger steak | Cream stew  **Black bean**  Cheese-in-hamburger steak | Hashed beef  **Fruit granola**  Fried chicken | Beef curry  **Sweet potato**  Hamburger steak |
| 15-8  / 17-8 | Butter chicken curry  **No snack**  Fried chicken | Beef curry  **Black bean**  Hamburger steak | Cream stew  **Fruit granola**  Cheese-in-hamburger steak | Hashed beef  **Sweet potato**  Fried chicken | Beef curry  **Potato chips**  Hamburger steak |
| 15-9  / 17-9 | Butter chicken curry  **Black bean**  Fried chicken | Beef curry  **Fruit granola**  Hamburger steak | Cream stew  **Sweet potato**  Cheese-in-hamburger steak | Hashed beef  **Potato chips**  Fried chicken | Beef curry  **No snack**  Hamburger steak |
| 15-10  / 17-10 | Butter chicken curry  **Fruit granola**  Fried chicken | Beef curry  **Sweet potato**  Hamburger steak | Cream stew  **Potato chips**  Cheese-in-hamburger steak | Hashed beef  **No snack**  Fried chicken | Beef curry  **Black bean**  Hamburger steak |
| 15-11  / 17-11 | Hashed beef  **Sweet potato**  Hamburger steak | Butter chicken curry  **Potato chips**  Cheese-in-hamburger steak | Beef curry  **No snack**  Fried chicken | Cream stew  **Black bean**  Hamburger steak | Hashed beef  **Fruit granola**  Cheese-in-hamburger steak |
| 15-12  / 17-12 | Hashed beef  **Potato chips**  Hamburger steak | Butter chicken curry  **No snack**  Cheese-in-hamburger steak | Beef curry  **Black bean**  Fried chicken | Cream stew  **Fruit granola**  Hamburger steak | Hashed beef  **Sweet potato**  Cheese-in-hamburger steak |
| 15-13  / 17-13 | Hashed beef  **No snack**  Hamburger steak | Butter chicken curry  **Black bean**  Cheese-in-hamburger steak | Beef curry  **Fruit granola**  Fried chicken | Cream stew  **Sweet potato**  Hamburger steak | Hashed beef  **Potato chips**  Cheese-in-hamburger steak |
| 15-14  / 17-14 | Hashed beef  **Black bean**  Hamburger steak | Butter chicken curry  **Fruit granola**  Cheese-in-hamburger steak | Beef curry  **Sweet potato**  Fried chicken | Cream stew  **Potato chips**  Hamburger steak | Hashed beef  **No snack**  Cheese-in-hamburger steak |
| 15-15  / 17-15 | Hashed beef  **Fruit granola**  Hamburger steak | Butter chicken curry  **Sweet potato**  Cheese-in-hamburger steak | Beef curry  **Potato chips**  Fried chicken | Cream stew  **No snack**  Hamburger steak | Hashed beef  **Black bean**  Cheese-in-hamburger steak |
| 15-16  / 17-16 | Butter chicken curry  **Sweet potato**  Fried chicken | Beef curry  **Potato chips**  Hamburger steak | Cream stew  **No snack**  Cheese-in-hamburger steak | Hashed beef  **Black bean**  Fried chicken | Beef curry  **Fruit granola**  Hamburger steak |
| 15-17  / 17-17 | Butter chicken curry  **Potato chips**  Fried chicken | Beef curry  **No snack**  Hamburger steak | Cream stew  **Black bean**  Cheese-in-hamburger steak | Hashed beef  **Fruit granola**  Fried chicken | Beef curry  **Sweet potato**  Hamburger steak |
| 15-18  / 17-18 | Butter chicken curry  **No snack**  Fried chicken | Beef curry  **Black bean**  Hamburger steak | Cream stew  **Fruit granola**  Cheese-in-hamburger steak | Hashed beef  **Sweet potato**  Fried chicken | Beef curry  **Potato chips**  Hamburger steak |
| 15-19  / 17-19 | Butter chicken curry  **Black bean**  Fried chicken | Beef curry  **Fruit granola**  Hamburger steak | Cream stew  **Sweet potato**  Cheese-in-hamburger steak | Hashed beef  **Potato chips**  Fried chicken | Beef curry  **No snack**  Hamburger steak |
| 15-20  / 17-20 | Butter chicken curry  **Fruit granola**  Fried chicken | Beef curry  **Sweet potato**  Hamburger steak | Cream stew  **Potato chips**  Cheese-in-hamburger steak | Hashed beef  **No snack**  Fried chicken | Beef curry  **Black bean**  Hamburger steak |

Top; Lunch contents, Middle; Snack contents (**bold**), Bottom; Dinner contens.

**Supplemental Table S2. Nutrition information for lunch.**

| Lunch Contents | Hashed beef | Butter chicken curry | Beef curry | Cream stew |
| --- | --- | --- | --- | --- |
|  | Bread 6 slices  Hashed beef  Minestrone | Bread 6 slices  Butter Chicken Curry  Corn Soup | Bread 6 slices  Beef curry  Minestrone | Bread 6 slices  Cream stew  corn soup |
| Energy (kJ) | 2015/2723 | 2153/2861 | 2057/2765 | 2090/2798 |
| Protein (g) | 17.2/23.1 | 15.2/21.1 | 16.6/22.5 | 20.9/26.8 |
| Fat (g) | 17.1/19.7 | 23.2/25.8 | 19.1/21.7 | 21.1/23.7 |
| Sugars (g) | 64.8/95.4 | 60.1/90.7 | 63.4/94.0 | 55.5/86.1 |

Women eat one piece of bread and men eat two pieces of bread. The difference in caloric intake between men and women was compensated for by the number of pieces of bread eaten.

**Supplemental Table S3. Nutrition information for dinner.**

| Dinner Contents | Hamburger steak | Cheese-in-hamburger steak | Fried chicken |
| --- | --- | --- | --- |
|  | Rice, hamburger steak with demi-glace sauce, sauteed onions, omelet with tomato sauce, mashed potatoes, sauteed spinach and corn, broccoli, miso soup. | Rice, Cheese-in-hamburger steak, broccoli, sauteed spinach and bacon, potato salad, edamame and egg salad, vegetables in tomato sauce, corn soup | Rice, fried chicken, broccoli, sauteed konnyaku and carrot, okra and corn salad, fried Chinese cabbage and shiitake mushroom with starchy sauce, boiled spinach, cooked vegetables, miso soup |
| Energy (kJ) | 2622 | 2673 | 2673 |
| Protein (g) | 21.9 | 20.8 | 21.1 |
| Fat (g) | 15.9 | 18.2 | 19 |
| Total Carbohydrates (g) | 94.1 | 93.0 | 90.6 |
| - Sugars (g) | 87.5 | 82.6 | 83.6 |
| - Dietary fiber (g) | 3.4 | 4.6 | 3.4 |

**Supplemental Table S4. Values of monitoring glucose level at 15:00 snack.**

| Time | NO | BB | PC | FG | SP |
| --- | --- | --- | --- | --- | --- |
| 15:00 | 91.5 ± 18.3 | 91.3 ± 14.5 | 89.8 ± 11.2 | 90.9 ± 15.8 | 86.8 ± 12.8 |
| 15:15 | 88.5 ± 20.0 | 85.4 ± 11.3 | 86.9 ± 10.7 | 90.9 ± 14.7 | 91.5 ± 12.7 |
| 15:30 | 84.6 ± 17.3 | 82.1 ± 12.9 | 88.4 ± 12.6 | 100.1 ± 21.1 | 106.2 ± 18.7* |
| 15:45 | 81.6 ± 13.3 | 80.0 ± 14.0 | 90.2 ± 12.8 | 108.4 ± 17.2** | 117.4 ± 20.4** |
| 16:00 | 80.9 ± 12.8 | 77.9 ± 10.3 | 91.9 ± 12.7 | 108.1 ± 12.6** | 116.2 ± 19.3** |
| 16:15 | 81.2 ± 12.8 | 77.9 ± 7.7 | 92.2 ± 13.2 | 101.8 ± 17.7** | 111.8 ± 20.7** |
| 16:30 | 80.9 ± 12.4 | 79.6 ± 7.6 | 90.9 ± 13.2 | 94.4 ± 17.7* | 108.8 ± 19.8** |
| 16:45 | 81.2 ± 12.4 | 82.1 ± 10.7 | 90.2 ± 15.2 | 88.3 ± 13.9 | 103.8 ± 17.2** |
| 17:00 | 81.5 ± 11.8 | 83.1 ± 12.2 | 88.3 ± 15.8 | 83.9 ± 13.5 | 97.6 ± 15.6* |
| 17:15 | 81.2 ± 11.0 | 82.9 ± 10.2 | 83.6 ± 11.1 | 81.2 ± 13.9 | 91.5 ± 16.0 |
| 17:30 | 82.5 ± 11.3 | 82.9 ± 8.2 | 79.8 ± 8.9 | 79.1 ± 12.7 | 85.4 ± 16.2 |
| 17:45 | 83.2 ± 10.6 | 82.8 ± 8.1 | 78.6 ± 7.4 | 77.8 ± 11.3 | 80.7 ± 14.8 |
| 18:00 | 82.4 ± 9.2 | 82.8 ± 9.0 | 79.6 ± 7.4 | 78.2 ± 10.1 | 75.3 ± 10.9* |
| 18:15 | 82.3 ± 8.8 | 82.8 ± 8.9 | 80.8 ± 7.9 | 78.0 ± 9.2 | 72.6 ± 10.3** |
| 18:30 | 81.9 ± 8.0 | 83.2 ± 8.1 | 81.1 ± 8.6 | 77.1 ± 9.2 | 72.7 ± 10.9** |
| 18:45 | 80.6 ± 7.8 | 83.8 ± 8.0 | 80.2 ± 8.7 | 77.1 ± 9.2 | 74.2 ± 8.9** |
| 19:00 | 81.2 ± 7.3 | 83.4 ± 8.2 | 80.6 ± 8.4 | 77.8 ± 9.0 | 75.9 ± 7.7* |
| 19:15 | 93.6 ± 14.0 | 91.9 ± 10.9 | 91.2 ± 7.3 | 88.2 ± 8.3 | 86.9 ± 11.1 |
| 19:30 | 122.4 ± 15.5 | 116.0 ± 23.8 | 120.2 ± 18.5 | 114.7 ± 16.3 | 112.6 ± 19.4 |
| 19:45 | 147.4 ± 19.9 | 138.1 ± 35.4 | 142.7 ± 24.3 | 135.6 ± 22.1 | 129.0 ± 26.3* |
| 20:00 | 153.9 ± 27.0 | 145.8 ± 33.8 | 142.8 ± 23.0 | 132.6 ± 22.0** | 122.1 ± 25.5** |
| 20:15 | 150.9 ± 27.5 | 145.7 ± 28.1 | 135.4 ± 22.6 | 121.1 ± 22.3** | 111.3 ± 20.1** |
| 20:30 | 145.4 ± 29.0 | 143.0 ± 28.2 | 130.1 ± 23.3 | 115.4 ± 21.9** | 109.9 ± 20.8** |
| 20:45 | 139.1 ± 28.6 | 139.0 ± 20.5 | 127.1 ± 22.6 | 118.4 ± 21.4** | 110.8 ± 17.0** |
| 21:00 | 131.6 ± 28.1 | 135.4 ± 32.1 | 124.8 ± 21.2 | 117.9 ± 19.0 | 107.8 ± 14.6** |
| 21:15 | 124.8 ± 23.6 | 127.7 ± 36.1 | 121.4 ± 17.2 | 112.8 ± 18.1 | 104.2 ± 13.1** |
| 21:30 | 118.1 ± 16.0 | 119.8 ± 32.8 | 112.2 ± 14.2 | 107.9 ± 18.6 | 103.8 ± 12.5** |
| 21:45 | 107.5 ± 15.2 | 113.6 ± 25.6 | 105.6 ± 16.3 | 103.9 ± 18.7 | 104.8 ± 12.1 |
| 22:00 | 98.9 ± 14.8 | 109.1 ± 23.2 | 103.2 ± 18.8 | 100.6 ± 17.4 | 104.6 ± 11.7 |
| 22:15 | 97.6 ± 16.8 | 105.5 ± 25.6 | 101.5 ± 17.9 | 98.5 ± 14.8 | 100.4 ± 11.9 |
| 22:30 | 99.3 ± 19.1 | 102.6 ± 20.4 | 98.4 ± 14.6 | 96.2 ± 15.5 | 95.5 ± 11.4 |
| 22:45 | 96.1 ± 18.5 | 99.1 ± 17.3 | 93.4 ± 13.3 | 94.1 ± 15.9 | 91.7 ± 11.4 |
| 23:00 | 91.5 ± 16.7 | 95.8 ± 15.9 | 89.1 ± 13.4 | 91.7 ± 12.6 | 88.9 ± 11.9 |
| 23:15 | 90.2 ± 13.5 | 93.2 ± 15.0 | 86.5 ± 12.3 | 85.8 ± 11.1 | 88.8 ± 11.7 |
| 23:30 | 87.6 ± 8.0 | 90.6 ± 12.9 | 84.5 ± 11.4 | 81.8 ± 11.9 | 88.2 ± 11.5 |
| 23:45 | 83.4 ± 8.1 | 87.1 ± 11.6 | 83.1 ± 12.5 | 81.8 ± 13.1 | 86.5 ± 11.2 |
| 24:00 | 80.9 ± 9.5 | 84.5 ± 11.4 | 82.7 ± 14.0 | 82.4 ± 13.6 | 85.5 ± 12.3 |

Glucose (mg/dL) was measured by FreeStyle Liber Pro. Group NO; 15:00 no-snack, 19:00 dinner. Group BB; 15:00 fried black beans snack, 19:00 dinner. Group PC; 15:00 potato chips snack, 19:00 dinner. Group FG; 15:00 fruit granola snack, 19:00 dinner. Group SP; 15:00 roasted sweet potato snack, 19:00 dinner. Values are means ± standard deviation. Two way-repeated-ANOVA (Time; F = 50.80, *p* <0.01, Group; F = 0.83, *p* = 0.47, Interaction; F = 8.77, *p* < 0.01.) was used for glucose data, and Dunnett's was used for multiple testing vs NO group, * *p* < 0.05, ** *p* < 0.01.

**Supplemental Table S5. Values of glucose response at 15:00 snack.**

|  | Analysis | NO | BB | PC | FG | SP | F value, p value |
| --- | --- | --- | --- | --- | --- | --- | --- |
| Snack | Maximal glucose (mg/dL) | 93.9 ± 17.7 | 96.8 ± 14.8 | 103.7 ± 10.4 | 119.0 ± 12.3** | 130.7 ± 16.4** | F = 21.72,  *p* < 0.01 |
|  | Δ Maximal glucose (mg/dL) | 2.5 ± 3.9 | 5.5 ± 9.1 | 13.9 ± 11.5** | 28.1 ± 20.3** | 43.9 ± 22.6** | F = 26.46,  *p* < 0.01 |
|  | AUC (min*mg/dL) | 9982 ± 1602 | 9783 ± 796 | 10798 ± 1090 | 11690 ± 1113** | 12717 ± 925** | F = 25.24,  *p* < 0.01 |
|  | IAUC (min*mg/dL) | 109 ± 228 | 172 ± 271 | 605 ± 721* | 1238 ± 1086*** | 2440 ± 1484** | F = 24.23,  *p* < 0.01 |
| Dinner | Maximal glucose (mg/dL) | 164.6 ± 20.0 | 158.7 ± 29.8 | 149.4 ± 22.4* | 142.9 ± 18.7** | 138.1 ± 18.1** | F = 8.39,  *p* < 0.01 |
|  | Δ Maximal glucose (mg/dL) | 83.5 ± 23.6 | 75.8 ± 29.2 | 68.8 ± 25.0 | 65.1 ± 15.7** | 62.2 ± 14.5** | F = 5.28,  *p* < 0.01 |
|  | AUC (min*mg/dL) | 33903 ± 2689 | 34241 ± 4567 | 32620 ± 3400 | 31171 ± 3332** | 30426 ± 2488** | F = 8.34,  *p* < 0.01 |
|  | IAUC (min*mg/dL) | 9638 ± 3761 | 9698 ± 4631 | 8666 ± 3524 | 7995 ± 2788 | 7678 ± 2721 | F = 2.97,  *p* < 0.05 |

Values are means ± standard deviation. One way-repeated-ANOVA was used and Dunnett's was used for multiple testing vs NO group, * *p* < 0.05, ** *p* < 0.01. NO, no-snack; BB, fried black beans snack; PC, potato chips snack; FG, fruit granola snack; SP, roasted sweet potato snack; AUC, area under the curve of glucose; IAUC, incremental area under the curve of glucose.

**Supplemental Table S6. Values of monitoring glucose level at 17:00 p.m. snack.**

| Time | NO | BB | PC | FG | SP |
| --- | --- | --- | --- | --- | --- |
| 17:00 | 73.8 ± 9.1 | 78.4 ± 9.3 | 76.5 ± 16.4 | 79.3 ± 7.8 | 82.1 ± 19.2 |
| 17:15 | 75.1± 8.7 | 78.2 ± 7.7 | 81.6 ± 18.7 | 88.3 ± 8.5** | 91.8 ± 21.4** |
| 17:30 | 76.6 ± 9.3 | 79.0 ± 5.9 | 93.2 ± 18.2** | 111.6 ± 17.4** | 116.8 ± 22.8** |
| 17:45 | 77.3 ± 10.7 | 81.8 ± 5.2 | 99.9 ± 18.4** | 130.9 ± 18.4** | 142.6 ± 25.6** |
| 18:00 | 76.9 ± 10.4 | 83.4 ± 5.8* | 100.6 ± 16.9** | 129.2 ± 16.8** | 146.3 ± 30.3** |
| 18:15 | 76.8 ± 8.6 | 84.1 ± 6.2** | 98.0 ± 17.1** | 114.2 ± 17.5** | 135.6 ± 26.0** |
| 18:30 | 78.7 ± 8.0 | 85.3 ± 6.7** | 91.6 ± 14.1* | 102.3 ± 18.1** | 125.9 ± 23.4** |
| 18:45 | 81.3 ± 11.9 | 86.9 ± 10.2** | 85.3 ± 12.3 | 96.5 ± 15.7** | 115.5 ± 25.8** |
| 19:00 | 84.5 ± 19.5 | 91.2 ± 20.1** | 85.0 ± 17.6 | 90.9 ± 13.8** | 104.3 ± 25.0* |
| 19:15 | 93.3 ± 26.2 | 99.6 ± 24.9 | 92.4 ± 20.0 | 91.9 ± 19.4 | 97.8 ± 24.8 |
| 19:30 | 117.1 ± 30.5 | 112.4 ± 24.3 | 108.1 ± 22.9 | 105.1 ± 25.4 | 103.9 ± 20.0 |
| 19:45 | 146.0 ± 29.4 | 124.9 ± 21.9** | 121.1 ± 32.5* | 119.4 ± 24.1** | 111.8 ± 15.3** |
| 20:00 | 156.9 ± 27.8 | 133.1 ± 25.7** | 119.2 ± 34.1** | 120.8 ± 25.5** | 109.6 ± 19.5** |
| 20:15 | 150.0 ± 29.1 | 134.3 ± 32.0* | 111.1 ± 28.3** | 111.9 ± 23.1** | 106.2 ± 19.2** |
| 20:30 | 141.4 ± 37.7 | 131.1 ± 32.5 | 107.2 ± 24.1** | 104.8 ± 20.5** | 104.2 ± 18.6** |
| 20:45 | 135.3 ± 39.0 | 129.0 ± 30.2 | 107.9 ± 23.5* | 104.7 ± 20.2** | 100.5 ± 18.6** |
| 21:00 | 128.9 ± 34.9 | 126.1 ± 24.5 | 110.2 ± 21.9 | 106.8 ± 20.0* | 97.8 ± 11.4** |
| 21:15 | 123.2 ± 34.2 | 122.2 ± 18.4 | 110.9 ± 18.1 | 107.3 ± 19.2 | 95.9 ± 12.2* |
| 21:30 | 116.8 ± 30.6 | 118.1 ± 18.2 | 104.8 ± 19.6.6 | 106.8 ± 18.8 | 95.1 ± 15.3* |
| 21:45 | 109.9 ± 23.6 | 112.3 ± 18.9 | 98.3 ± 18.6 | 103.9 ± 16.4 | 94.9 ± 14.6 |
| 22:00 | 102.6 ± 17.0 | 106.4 ± 20.2 | 95.1 ± 17.6 | 99.7 ± 18.5 | 92.4 ± 14.3 |
| 22:15 | 95.3 ± 15.4 | 100.5 ± 18.7 | 92.6 ± 15.5 | 95.4 ± 17.4 | 90.4 ± 13.7 |
| 22:30 | 91.7 ± 15.4 | 95.7 ± 14.4 | 90.6 ± 16.0 | 90.9 ± 16.2 | 88.9 ± 13.0 |
| 22:45 | 90.2 ± 13.4 | 92.9 ± 11.9 | 90.1 ± 15.0 | 89.4 ± 14.1 | 86.2 ± 11.3 |
| 23:00 | 87.3 ± 11.6 | 92.1 ± 12.3 | 89.3 ± 14.9 | 90.3 ± 11.9 | 84.8 ± 9.7 |
| 23:15 | 83.2 ± 10.5 | 91.3 ± 14.6 | 89.1 ± 14.3 | 89.1 ± 12.5 | 84.1 ± 8.8 |
| 23:30 | 80.2 ± 10.2 | 88.1 ± 14.4 | 88.1 ± 13.1 | 87.4 ± 11.6 | 82.8 ± 8.5 |
| 23:45 | 78.7 ± 10.1 | 83.6 ± 11.0 | 85.7 ± 12.3 | 86.1 ± 11.4 | 81.4 ± 9.3 |
| 24:00 | 77.4 ± 8.3 | 80.2 ± 10.0 | 84.0 ± 12.8 | 85.2 ± 12.9 | 80.3 ± 8.5 |

Glucose (mg/dL) was measured by FreeStyle Liber Pro. Group NO; 17:00 no-snack, 19:00 dinner. Group BB; 17:00 fried black beans snack, 19:00 dinner. Group PC; 17:00 potato chips snack, 19:00 dinner. Group FG; 17:00 fruit granola snack, 19:00 dinner. Group SP; 17:00 roasted sweet potato snack, 19:00 dinner. Values are means ± standard deviation. Two way-repeated-ANOVA (Time; F = 22.26, *p* <0.01, Group; F = 2.42, *p* = 0.08, Interaction; F _(7.016, 126.3)_ = 17.22, *p* < 0.01.) was used for glucose data, and Dunnett's was used for multiple testing vs NO group, * *p* < 0.05, ** *p* < 0.01.

**Supplemental Table S7. Values of glucose response at 17:00 snack.**

|  | Analysis | NO | BB | PC | FG | SP | F value, p value |
| --- | --- | --- | --- | --- | --- | --- | --- |
| Snack | Maximal glucose (mg/dL) | 82.4 ± 7.7 | 88.1 ± 6.9** | 112.8 ± 13.6** | 135.9 ± 16.7** | 158.7 ± 22.7** | F = 91.89,  p < 0.01 |
|  | Δ Maximal glucose (mg/dL) | 12.1 ± 15.9 | 14.3 ± 17.9 | 36.4 ± 12.1** | 56.7 ± 17.8** | 76.6 ± 31.4** | F = 28.25,  p < 0.01 |
|  | AUC (min*mg/dL) | 9327 ± 1139 | 9952 ± 796* | 10965 ± 1315** | 12870 ± 1185** | 14513 ± 1510** | F = 77.42,  p < 0.01 |
|  | IAUC (min*mg/dL) | 466 ± 367 | 615 ± 542 | 1959 ± 866** | 3371 ± 1392*** | 4823 ± 2137** | F = 38.60,  p < 0.01 |
| Dinner | Maximal glucose (mg/dL) | 165.0 ± 29.4 | 149.3 ± 21.8** | 139.6 ± 18.7** | 135.4 ± 21.1** | 127.8 ± 21.3** | F = 13.90,  p < 0.01 |
|  | Δ Maximal glucose (mg/dL) | 86.3 ± 28.8 | 62.8 ± 21.5** | 51.9 ± 20.6** | 46.3 ± 15.0** | 29.8 ± 14.7** | F = 31.53,  p < 0.01 |
|  | AUC (min*mg/dL) | 33132 ± 4637 | 32689 ± 3596 | 29944 ± 3769** | 29995 ± 3652** | 28514 ± 2574** | F = 16.43,  p < 0.01 |
|  | IAUC (min*mg/dL) | 9842 ± 4323 | 7063 ± 3260* | 5025 ± 3392** | 4029 ± 2247** | 2231 ± 1796** | F = 26.77,  p < 0.01 |

Values are means ± standard deviation. One way-repeated-ANOVA was used and Dunnett's was used for multiple testing vs NO group, * *p* < 0.05, ** *p* < 0.01. NO, no-snack; BB, fried black beans snack; PC, potato chips snack; FG, fruit granola snack; SP, roasted sweet potato snack; AUC, area under the curve of glucose; IAUC, incremental area under the curve of glucose.

**Supplemental Table S8. Values of total AUC.**

| Study | NO | BB | PC | FG | SP |
| --- | --- | --- | --- | --- | --- |
| 15:00 snack | 43171 ± 2998 | 43097 ± 4784 | 42755 ± 3576 | 43420 ± 3245 | 42623 ± 3178 |
| 17:00 snack | 42458 ± 5249 | 42641 ± 4102 | 40909 ± 4466 | 42866 ± 4162 | 43027 ± 3485 |

Values are means ± standard deviation. One way-repeated-ANOVA was used and Dunnett's was used for multiple testing vs NO group. NO, no-snack; BB, fried black beans snack; PC, potato chips snack; FG, fruit granola snack; SP, roasted sweet potato snack.

**Supplemental Table S9. Values of amplitude range of glucose.**

| Study | NO | BB | PC | FG | SP |
| --- | --- | --- | --- | --- | --- |
| 15:00 snack | 93.4 ± 23.3 | 88.6 ± 30.8 | 77.0 ± 24.9 | 72.8 ± 17.3** | 72.5 ± 15.7** |
| 17:00 snack | 96.2 ± 30.4 | 76.1 ± 23.1 | 69.6 ± 15.3* | 70.8 ± 17.4* | 87.7 ± 22.3 |

Values are means ± standard deviation. Friedman test was used and Dunn's was used for multiple testing vs NO group, * *p* < 0.05, ** *p* < 0.01. NO, no-snack; BB, fried black beans snack; PC, potato chips snack; FG, fruit granola snack; SP, roasted sweet potato snack.

**Supplemental Table S10. Values of distribution of glucose at 15:00 snack.**

|  | NO | BB | PC | FG | SP |
| --- | --- | --- | --- | --- | --- |
| < 70 mg/dL | 41.5 ± 77.1 | 22.1 ± 31.0 | 17.6 ± 47.5 | 31.8 ± 73.1 | 41.5 ± 41.9 |
| 70 – 140 mg/dL | 598.2 ± 113.7 | 611.5 ± 89.0 | 637.9 ± 92.6 | 642.4 ± 76.0 | 645.9 ± 53.7 |
| > 140 mg/dL | 80.3 ± 56.9 | 86.5 ± 71.6 | 64.4 ± 59.2 | 45.9 ± 40.6 | 32.6 ± 39.8* |

Values of time (min) are means ± standard deviation. Friedman test was used and Dunn's was used for multiple testing vs NO group, * *p* < 0.05, ** *p* < 0.01. NO, no-snack; BB, fried black beans snack; PC, potato chips snack; FG, fruit granola snack; SP, roasted sweet potato snack.

**Supplemental Table S11. Values of distribution of glucose at 17:00 snack.**

|  | NO | BB | PC | FG | SP |
| --- | --- | --- | --- | --- | --- |
| < 70 mg/dL | 55.3 ± 80.4 | 38.4 ± 89.5 | 67.5 ± 84.5 | 23.4 ± 55.6* | 37.5 ± 53.9 |
| 70 – 140 mg/dL | 580.3 ± 113.5 | 617.8 ± 100.3 | 616.9 ± 80.7 | 655.3 ± 74.6* | 628.1 ± 73.7 |
| > 140 mg/dL | 84.4 ± 73.0 | 63.8 ± 70.9 | 35.6 ± 41.7** | 41.3 ± 42.6 | 52.5 ± 39.1 |

Values of time (min) are means ± standard deviation. Friedman test was used and Dunn's was used for multiple testing vs NO group, * *p* < 0.05, ** *p* < 0.01. NO, no-snack; BB, fried black beans snack; PC, potato chips snack; FG, fruit granola snack; SP, roasted sweet potato snack.
